# Microglial repopulation reverses radiation-induced cognitive dysfunction by restoring medial prefrontal cortex activity and modulating leukotriene-C4 synthesis

**DOI:** 10.1186/s40478-025-02026-8

**Published:** 2025-05-19

**Authors:** Yubo Hu, Zhe Li, Yafeng Zhu, Mengdan Xing, Xiaoru Xie, Panwu Zhao, Xin Cheng, Chuan Xiao, Yuting Xia, Jingru Wu, Yuan Luo, Ho Ko, Yamei Tang, Xiaojing Ye, Wei-Jye Lin

**Affiliations:** 1 Medical College of Jiaying University, Meizhou, Guangdong 514031 China; 2 Faculty of Forensic Medicine, Zhongshan School of Medicine, Sun Yat-sen University, Guangzhou, Guangdong 510080 China; 3 Guangdong Province Translational Forensic Medicine Engineering Technology Research Center, Sun Yat-sen University, Guangzhou, Guangdong 510080 China; 4 Guangdong Province Key Laboratory of Brain Function and Disease, Zhongshan School of Medicine, Sun Yat-sen University, Guangzhou, Guangdong 510080 China; 5 Guangdong Provincial Key Laboratory of Malignant Tumor Epigenetics and Gene Regulation, Guangdong-Hong Kong Joint Laboratory for RNA Medicine, Medical Research Center, Sun Yat-sen Memorial Hospital, Sun Yat-sen University, Guangzhou, 510120 China; 6 Brain Research Center, Sun Yat-Sen Memorial Hospital, Sun Yat-Sen University, Guangzhou, 510120 China; 7 Department of Neurology, Sun Yat-sen Memorial Hospital, Sun Yat-sen University, Guangzhou, Guangdong 510080 China; 8 Nanhai Translational Innovation Center of Precision Immunology, Sun Yat-Sen Memorial Hospital, Foshan, 528200 China; 9 Division of Neurology, Department of Medicine and Therapeutics, Faculty of Medicine, The Chinese University of Hong Kong, SAR, Hong Kong, China; 10 Li Ka Shing Institute of Health Sciences, The Chinese University of Hong Kong, SAR, Hong Kong, China; 11 Department of Psychiatry, Faculty of Medicine, The Chinese University of Hong Kong, SAR, Hong Kong, China; 12 School of Biomedical Sciences, Faculty of Medicine, The Chinese University of Hong Kong, SAR, Hong Kong, China; 13 Gerald Choa Neuroscience Center, The Chinese University of Hong Kong, SAR, Hong Kong, China; 14 Margaret K. L. Cheung Research Centre for Management of Parkinsonism, Faculty of Medicine, The Chinese University of Hong Kong, SAR, Hong Kong, China; 15 Chow Yuk Ho Technology Center for Innovative Medicine, The Chinese University of Hong Kong, SAR, Hong Kong, China; 16 Peter Hung Pain Research Institute, Faculty of Medicine, The Chinese University of Hong Kong, SAR, Hong Kong, China

**Keywords:** Radiation-induced brain injury, Microglia, Medial prefrontal cortex, Neuroinflammation, Leukotriene-C4

## Abstract

Cranial radiotherapy and environmental radiation exposure are associated with increased risk of cognitive dysfunction, including memory deficits and mood disorders, yet the underlying mechanisms remain poorly understood. In this study, we demonstrate that cranial irradiation induces hypoactivity in the medial prefrontal cortex (mPFC) of mice, leading to anxiety-like behaviors and memory impairments, which can be prevented by optogenetic activation of mPFC excitatory neurons. Radiaiton exposure also causes a significant reduction in microglial density within the mPFC, accompanied by morphological and transcriptional alterations in the remaining microglia. Notably, microglial repopulation, achieved through CSF1R antagonist-mediated depletion prior to irradiation and subsequent repopulation, restores mPFC neuronal acitivity and reverses cognitive and behavioral deficits. Integrated bulk RNA sequencing and microglial proteomic analysis of the mPFC reveal that microglial repopulation specifically modulates the leukotriene-C4 biosynthesis pathway, without significant changes in canonical pro-inflammatory cytokines or chemokines. Importantly, pharmacological inhibition of leukotriene-C4 synthase ameliorates radiation-induced anxiety and memory impairments. These findings identify leukotriene-C4 signaling as a critical mechanism underlying radiation-induced cognitive dysfunction and suggest that microglial repopulation and targted inhibition of leukotriene-C4 represent potential therapeutic strategies for mitigating radiation-associated cognitive disorders.

**Supplementary Information:**

The online version contains supplementary material available at 10.1186/s40478-025-02026-8.

## Introduction

Cranial irradiation remains a primary treatment for head and neck cancers and cerebrovascular abnormalities. However, patients undergoing radiotherapy also face a significant risk of developing cognitive disorders [2, 35]. Radiation-induced mental disorders are also commonly observed in environments with heightened radiation levels, such as space, high-altitude areas, and specific industrial settings [67, 95, 99]. Studies reveal that over 37% of nasopharyngeal carcinoma survivors develop anxiety disorders following radiotherapy [68], with over 50% of oncology patients who survive more than six months following whole-brain radiation therapy experience permanent cognitive decline [50]. These cognitive and mood disturbances significantly hinder individual’s behavior and decision-making abilities, posing significant safety concerns. Nevertheless, our understandings of therapeutic options and the underlying pathological mechanisms toward radiation-induced brain injury, the brain regions impacted by radiation, and their roles in radiation-induced cognitive and mood disorders remain limited and largely unexplored.

Extensive research has implicated the involvement of microglia, the resident immune cells of the central nervous system, in the development of radiation-induced brain injury (RIBI) [76, 103, 115], centered mainly on radiation-induced release of pro-inflammatory cytokines such as COX-2, TNF-α, and prostaglandin E2 by activated microglia, which trigger neuroinflammatory responses and contribute to pathological brain damage [3, 42, 46, 102]. However, clinical observations reveal that patients with RIBI exhibit limited responses to anti-inflammatory treatments, suggesting that unknown mechanisms beyond the conventional corticosteroid- and COX-2-centered therapeies and neuroinflammatory perspective may be involved in the pathological and functional injury induced by crainial irradiation [20, 40].

Recent studies on microglial elimination and repopulation (microglial replenishment) represent a promising therapeutic strategy given the involvement and contribution of pathological microglia in aged brains and neurodegenerative diseases [27, 45, 61, 104]. Pharmacological inhibition of the colony stimulating factor 1 receptor (CSF1R) results in rapid depletion of microglia for more than 90% within 5–7 days, and release from CSF1R inhibition allows rapid expansion of microglia to the density similar to pre-depletion state, leading to the replacement of the microglial population [26, 110]. Microglial replenishment has been shown to protect hippocampus-dependent cognitive functions in mice subjected to neurodegenerative diseases or brain irradiation [22, 64, 102], potentially through eliminating or resetting neuroinflammation responses [58] or modifying neuronal plasticity [30]. However, the exact molecular mechanisms underlying the protective effect of microglial replenishment in RIBI remain largely unknown. It also remains unclear whether microglia participate in irradiation-induced cognitive impairments sorely through the pro-inflammatory effects of TNF-α, and IL-1β, and prostaglandin E2 [46, 102], or through the involvement of other unidentified pathological factors that are induced by radiation.

In this study, we demonstrated that whole brain cranial irradiation induced a decrease in neuronal activity of the medial prefrontal cortex (mPFC), accompanied by anxiety-like behaviors and memory deficits in mice, and a significant reduction in microglial density. Importantly, replenishing microglia, but not inhibiting neuroinflammation by minocycline, effectively mitigated radiation-induced neuronal hypoactivity in the mPFC. Mechanistically, RNA sequencing and proteomic data revealed that radiation induced genes linked to regulations of inflammatory response, proliferation and survivial of microglia, glycolysis, and the leukotriene C4 (LTC4) biosynthetic process in the mPFC. Furthermore, pharmacological inhibition of LTC4 synthesis restored mPFC activity and prevented radiation-induced cognitive impairments, underscoring the critical role of the LTC4 synthesis pathway in irradiation-induced brain dysfunction.

## Materials and methods

**Animals** All animal studies were approved by the Institutional Animal Care and Use Committee at Sun Yat-sen University. 2–3 months old female and male C57BL/6 mice were used. The mice were obtained from Laboratory Animal Center of Sun Yat-sen University (Guangzhou, China) and Guangdong Medical Laboratory Animal Center (Guangzhou, China). The mice were housed in groups of 4–5 in a specific pathogen-free animal facility with a 12 h light/dark cycle, 18 ~ 22 ℃ temperature and 50–60% humidity, and have access to food and water *ad libitum*.

**Cranial irradiation** Mice were anaesthetized with a mixture of tiletamine and zolazepam (Virbac, Alpes-Maritimes, France, 85 mg/kg, dissolved in 0.9% saline, i.p.) and xylazine (Huamu, Jilin, China, 10 mg/kg, dissolved in 0.9% saline, i.p.) [33], and placed in a 10 mm thick lead box with a 8 mm-wide opening on the top to expose the head of the mice for whole-brain cranial irradiation. An X-ray irradiator (RS2000, Rad Source, Buford, GA, USA) was used to deliver radiation. A single dose of 15 Gy irradiation was given at a rate of 3 Gy/min. The control mice in the sham group were subjected to the same anesthesia procedure but did not receive irradiation.

**Splash test** Mice were individually sprayed with a 10% sucrose solution onto their dorsal coat, placed in a familiar mouse cage, and videotaped for 5 min under a red light illumination and in a sound-insulated cabinet. The sucrose solution was used to initiate grooming behaviors in mice due to its viscosity. The time spent grooming was manually measured for the entire 5 min by an experimenter blinded to treatment groups. Previous studies have demonstrated that anxious mice exhibited increased grooming behavior [36].

**Light-dark box test** The light-dark box was composed of a dark compartment and a light compartment (12.5 × 12.5 cm^2^ each) illuminated with 600 lx white light, and connected by a door. Mice were individually placed into the dark compartment and allowed to explore both compartments freely for 10 min. The time spent in each compartment was recorded and measured by the DigBehv animal behavioral tracking and analyzing system (Ji-Liang, Shanghai, China) as described previously [12].

**Open field test** Mice were placed into a corner of an open field arena (40 × 40 cm) that was under red light illumination (90–120 lx), and videotaped for 5 min. The total travelled distance in the arena, as well as the distance and duration travelled in the center zone (20 × 20 cm) were measured by the DigBehv animal behavioral tracking and analyzing system (Ji-Liang, Shanghai, China) as described previously [48].

**Y maze** Y-maze was used to measure spatial working memory as described previously [62]. The Y-maze was consisted of 3 arms of 50 × 10 × 20 cm each. The mice were placed in the center zone of the maze and allowed to freely explore the maze for 5 min. An alternation event was defined as the completion of sequential entries into all three arms. Percent alternation (%Alteration) was calculated as follows: [(number of alternations)/(total entries − 2)] × 100.

**Immunofluorescence staining**,** imaging and image analysis** Mice were anesthetized and transcardially perfused with 4% paraformaldehyde in 1×PBS, and post-fixed with 4% paraformaldehyde overnight at 4 °C. 30 μm coronal cryosections were collected. Free-floating sections were blocked with 5% goat serum, 1% bovine serum albumin in 1×PBS with 0.4% Triton X-100 for 2 h at room temperature, then incubated with primary antibodies diluted in the blocking buffer for approximately 40 h at 4 °C. Afterwards, the sections were washed in 1×PBS with 0.4% Triton X-100, incubated with Alexa Fluor-conjugated secondary antibodies (Thermo Scientific, Waltham, MA) for 2 h at room temperature, washed again, and stained with DAPI (5 µg/mL, Cell Signaling Technologies, Danvers, MA, USA), before mounting on the glass slides. The primary antibodies used in this study included: (1) anti-c-FOS (1:750, cat# 2250, Cell Signaling Technologies, Danvers, MA, USA); (2) anti-CamKIIα (1:1000, cat# 05-532, Millipore, Burlington, MA, USA); (3) anti-GAD67 (1:3000, cat# MAB5406, Millipore, Burlington, MA, USA); (4) anti-NeuN (1:500, cat# 24307 S, Cell Signaling Technologies, Danvers, MA, USA); (5) anti-IBA1(1:500, cat# 019-19741, WAKO, Richmond, VA, USA); (6) anti-IBA1(1:500, cat# 234006, Synaptic Systems, Goettingen, Germany); (7) anti-CD68 (1:1000, cat# MCA1957GA, Bio-RAD, Hercules, California, USA); (8) anti-LTC4S (1:1000, cat# PA5121425, Thermo Fisher, Waltham, MA, USA).

For quantification of the density of c-FOS-positive cells: 20 brain regions were analyzed. For each region of interest, 2 or 3 brain slices were selected based on the anterior-posterior extent of the region and used for quantification. Both hemispheres were sampled. The data from each animal were averaged and presented as a single data point. Images were acquired by a Nikon Eclipse Ni-U epi-fluorescent microscope (Tokyo, Japan) at a 10× objective. Images were acquired and analyzed under consistent conditions for all samples. Regions of interests were defined manually by a researcher blinded to the experiment groups, according to the Paxinos and Franklin’s the Mouse Brain in Stereotaxic Coordinates (4th Edition, 2013) as well as the Allen brain reference atlas. c-FOS-positive nucleus were measured automatically in ImageJ (version 1.52p, NIH, US). Briefly, images were background subtracted and automatically thresholded. Particle size and circularity were determined by the Analyze Particle process to detect discrete c-FOS-positive nucleus without including background noise. The counts of c-FOS-positive nucleus were then divided by the area size of the region of interest. The raw densities of c-FOS + cells were first divided by the mean value of the sham group, and then the fold change over sham group were compared between the two groups. The statistical findings were presented as non-adjusted *p* value.

For quantification of the soma size, process length, branches points and sholl analysis of IBA1-positive microglia, images were taken using a Zeiss LSM780 confocal microscope (Jena, Germany) with a 40 × oil objective. 10 μm thick confocal z-stacks (optical section depth 0.67 μm, 15 sections / z stack) of 4 different fields of mPFC from the two hemispheres were acquired. The acquisition and analysis parameters were kept consistent for all the samples. The images were analyzed using iMARIS 10.0.1 software. Soma size, process length, branches points and sholl analysis of IBA-1-positive microglia were analyzed with the IMARIS function ‘‘Fliaments’’. Quantification of the fluorescence intensity of CD68 protein in IBA1-positive microglia were conducted using ImageJ software (Fiji edition, NIH) by an observer blinded to experimental groups. Data from each animal were averaged and presented as a data point.

**Optogenetics** Adeno-associated viruses (AAV) expressing channelrhodopsin-2 (ChR2) fused with mCherry, or mCherry alone, under the CaMK2α promoter (AAV8-CaMK2α-hChR2(E13A)-mCherry and AAV8-CaMK2α-mCherry) were made by OBiO (Shanghai, China). Mice were anesthetized with 5% isoflurane and maintained at 2% isoflurane during the surgery. Mouse head was fixed on a stereotactic frame (RWD Life Science, Shenzhen, China). Eyes of mice were lubricated with Vaseline, and their body temperature was maintained at 37 °C on a feedback-controlled heating pad (RWD Life Science, Shenzhen, China). The fur was shaved, and incision site was sterilized, after which an incision was made to expose the skull. A hole was drilled in the skull above the mPFC. A glass micropipette mounted onto a Nanoliter microinjector (World Precision Instruments, Sarasota, FL, USA) was lowered into the mPFC (1.8 mm anterior, 0.3 mm lateral from midline and 2.45 mm ventral to Bregma). 100 nL AAV (2 × 10^13^ v.g./mL) was microinjected at a rate of 50 nL/min. The glass micropipette was left in place for additional 5 min before slowly retracted, and the scalp was sutured. Cranial irradiation was performed 2 weeks after AAV injection. One week before the behavioral tests, an optic fiber (O.D. 1.25 mm, Thinker-Tech, Nanjing, China) was implanted in all mice, 50 μm above the AAV injection site through another stereotactic surgery. Dental cement was used to fix the optical fiber. Both AAV injection and fiber implantation were unilateral, and counter-balanced for the left and right mPFC. During subsequent splash test and open field test, we used the 3 min light-off– 3 min light-on– 3 min light-off paradigm. During the light-on phase, 20 Hz 3 mV 473 nm light with 10 ms pulse-width was delivered by a blue light laser (Thinker-Tech, Nanjing, China).

**RNA extraction and RNA-seq** Two weeks after 15 Gy cranial irradiation, the mice were cervically dislocated, and their brains were rapidly removed and sliced into 1 mm thick coronal sections using a brain matrix submerged in ice-cold dissection buffer. mPFC were punched out using a 15 G puncher and snap-frozen on dry ice. Total RNA was extracted using the Qiagen RNeasy Micro Kit (Hilden, Germany). RNA collected from the mPFC of 2–3 animals were pooled as one biological replicate. For the first RNA-seq experiment (see Fig. 3), 6 biological replicates for the sham group (Sham) and 7 replicates for the radiation group (RAD) were analyzed. For the second RNA-seq experiment (see Fig. 4), 4 biological replicates for the sham group (Sham), 4 replicates for the radiation + normal diet group (R + CON), and 3 replicates for the radiation + PLX3397 diet group (R + PLX) were analyzed. RNA samples were sent to MAGIGENE (Guangzhou, China) for quality control, cDNA library preparation, sequencing and read alignment. Samples with RNA integrity number (RIN) > 9 were used. Paired-end, 150 bp RNA sequencing was performed on an Illumina HiSeq system.

**Fig. 1:**
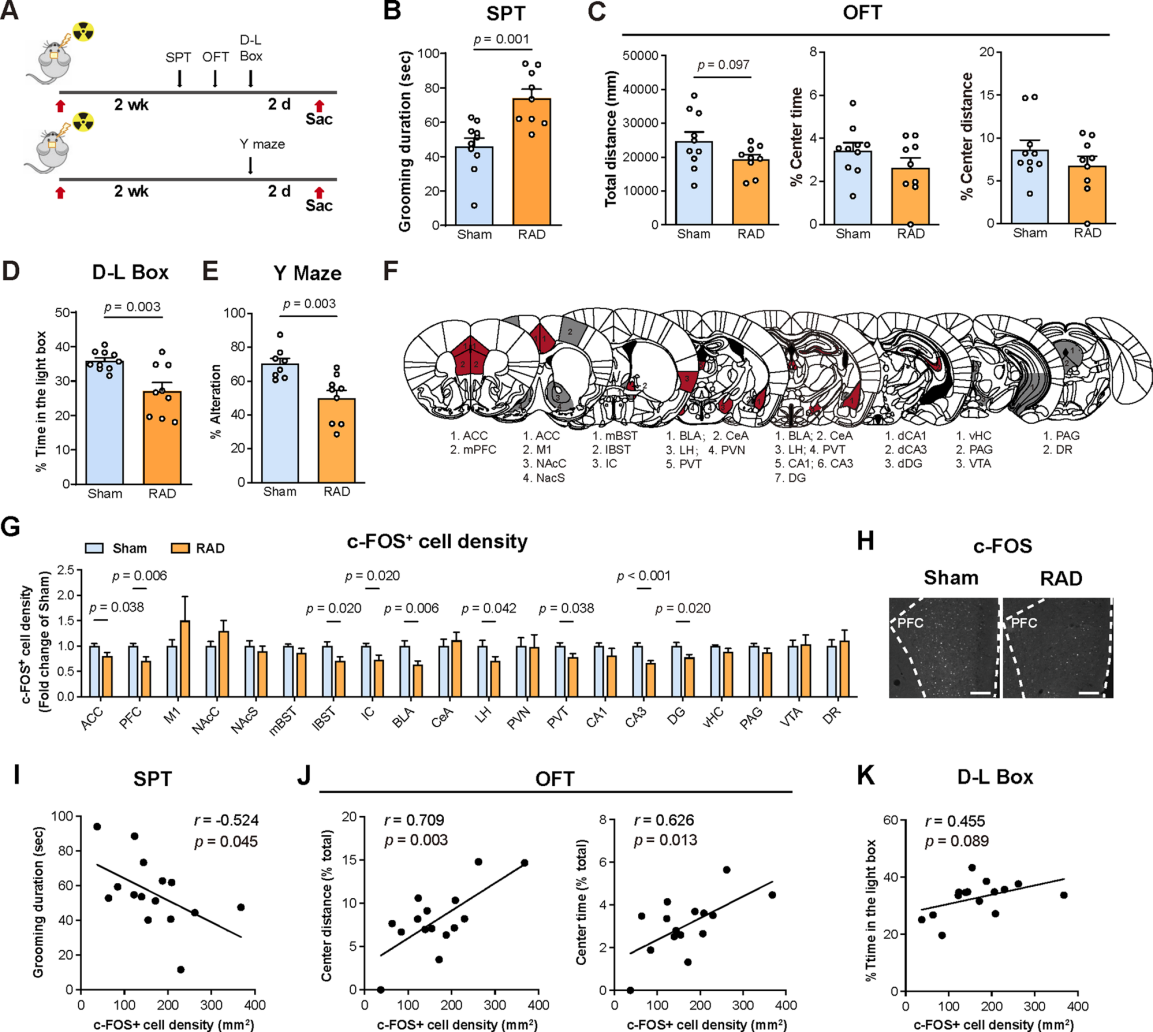
Decreased activity in the mPFC is associated with cranial irradiation induced anxiety-like behaviors. (**A**) Schematic of experiment procedures. Mice received sham irradiation (Sham) or 15 Gy cranial irradiation (RAD). Behavioral tests were conducted at 2 weeks (2 wk) post irradiation. Mice were sacrificed 2 days after the last behavioral test ended. SPT: splash test, OFT: open field test, D-L Box: dark-light box. (**B**) Quantification of the grooming duration during the splash test (Sham: *n* = 10; RAD: *n* = 9). (**C**) Quantification of total distance travelled, percentage of the time spent in center zone, and travelled distance in center zone as a percentage of the total distance in the open field test (Sham: *n* = 10; RAD: *n* = 9). (**D**) Quantification of time spend in the light box as a percentage of the total time in the D-L Box (Sham: *n* = 10; RAD: *n* = 9). (**E**) Quantification of % Alteration in the Y maze test (Sham: *n* = 9; RAD: *n* = 10). (**F**) The whole-brain analysis comprises 20 atlas-defined mouse brain regions. Colors reflect the decreased (red) or unaltered (gray) c-FOS expression in 15 Gy radiation group (RAD) compared to the sham radiation group (Sham). (**G**) The fold changes in the density of c-FOS positive cells in different brain regions, compare to the Sham group. Similar amounts of male and female mice were used for each group and the results were combined for statistical analysis (Sham: *n* = 13–15; RAD: *n* = 16–18; removed outliers: Sham-M1: *n* = 1; Sham-vHC: *n* = 1). (**H**) Representative images of c-FOS staining in the mPFC. Scale bar, 200 μm. (**I-K**) Correlation of the density of c-FOS positive cells in the mPFC with the grooming duration in the SPT (I), the percentage of travelled distance and time in the center zone compared to total distance and time (J), and the time spend in the light box as the percentage of total time in the D-L Box (K) (*n* = 15). Data are presented as mean ± s.e.m. and analyzed by student’s *t* test (B-E), multiple *t* tests (G) or Pearson’s correlation test (I-K)

**Fig. 2:**
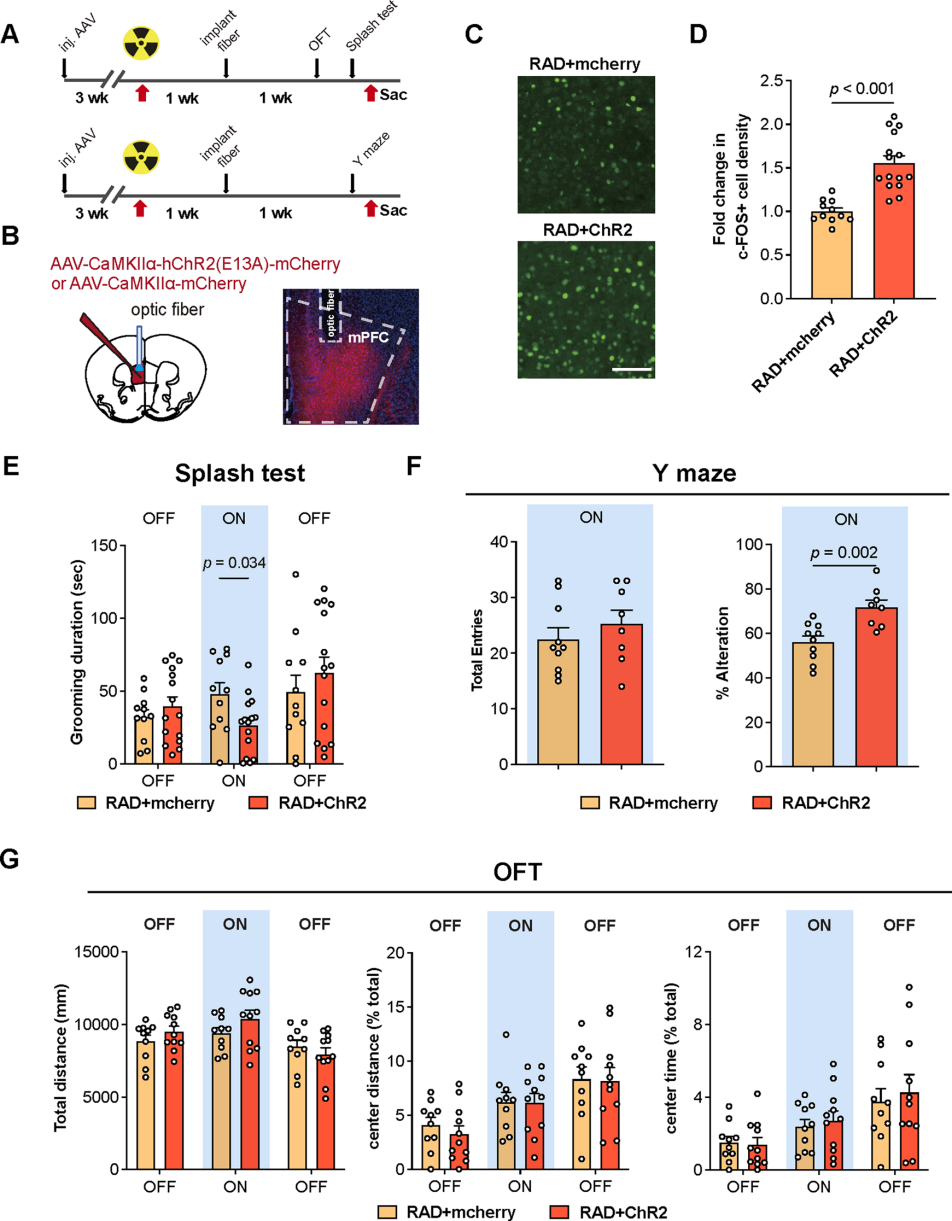
Optogenetic activation of excitatory neurons in the mPFC reduces radiation-induced anxiety-like behaviors and memory deficits. (**A**) Schematic of experimental procedures. AAVs were injected into the mPFC of mice 3 weeks before cranial irradiation. Optic fibers were implanted 1 week post irradiation and behavioral tests were performed 2 weeks post irradiation. (**B**) Illustrations of AAV injection strategy and a representative image of AAV-mediated mCherry expression and fiber implant position in the mPFC. (**C-D**) Representative images (C) and quantification of c-FOS positive neurons in the mPFC of the mCherry and ChR2 cohorts post irradiation (D, RAD + mCherry: *n* = 11; RAD + ChR2: *n* = 15). Scale bar: 100 μm. (**E**) Quantification of the grooming duration during light-off and light-on phases in the splash test (RAD + mCherry: *n* = 11; RAD + ChR2: *n* = 15). (**F**) Quantification of the total entries and the % Alteration in the Y maze test between groups (RAD + mCherry: *n* = 10; RAD + ChR2: *n* = 8). (**G**) Quantification of the total distance travelled, center distance travelled (as percentage of total moving distance), time spent in the center zone (as percentage of the total time) during light-off and light-on phases in the open field test (RAD + mCherry: *n* = 10; RAD + ChR2: *n* = 11; removed outliers: RAD + mCherry: *n* = 1, RAD + ChR2: *n* = 4). Similar amount of male and female mice was used for each group and the results were combined for statistical analysis. Data are presented as mean density ± s.e.m. and analyzed by Student’s *t* test (D, F) or two-way ANOVA with Fisher’s LSD *post hoc* test (E, G)

**Fig. 3:**
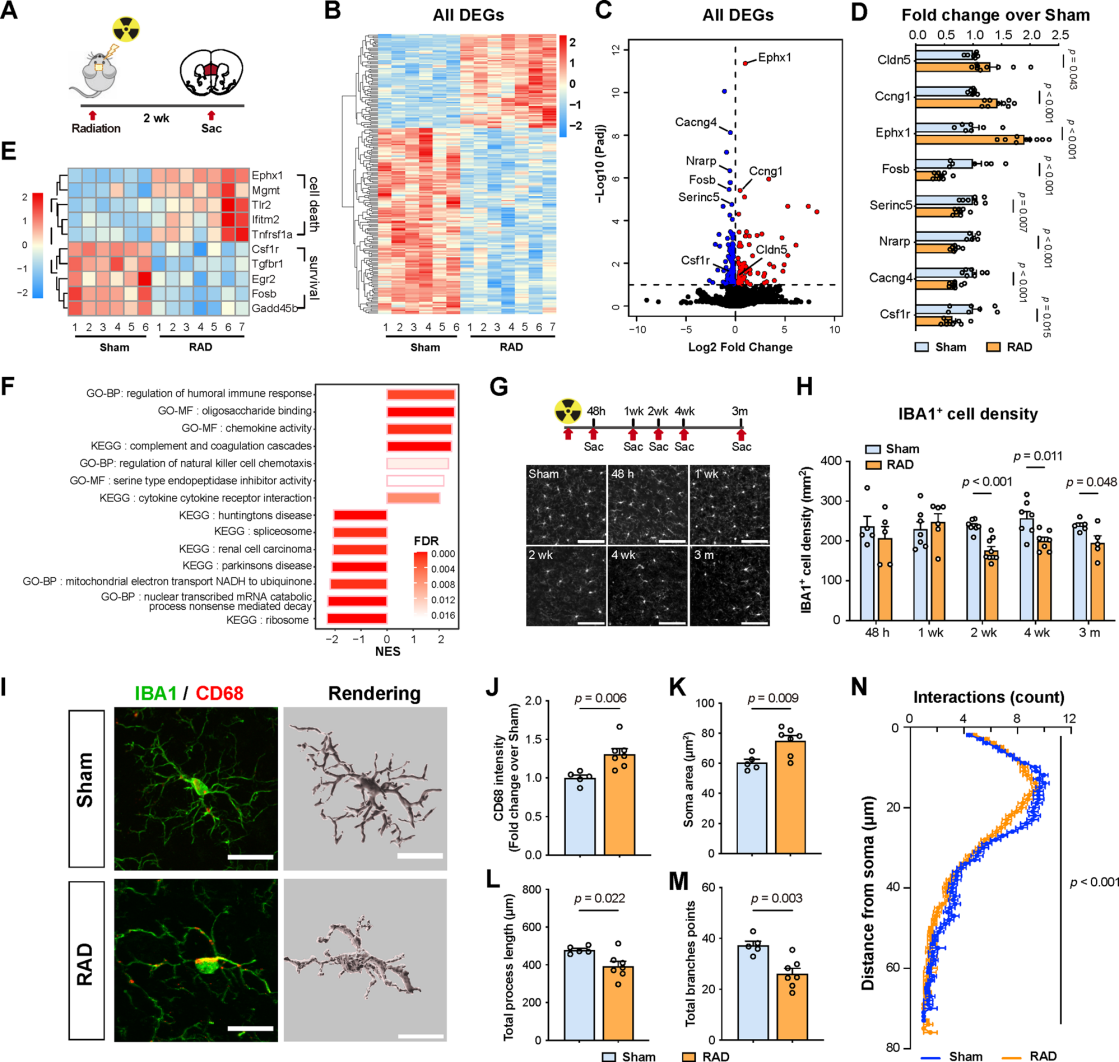
Radiation induces transcriptomic shifts and alterations in microglial density as well as properties in the mPFC. (**A**) Schematic of experimental procedures. The mPFC tissues were collected from mice at 2 weeks post irradiation (RAD, *n* = 3 for male, *n* = 4 for female), or non-irradiated control mice (Sham, *n* = 3 for male, *n* = 3 for female). (**B**) Heatmap showing relative expression levels of the differentially expressed genes (DEGs) (filtered by *p*-adjust < 0.1). (**C**) Volcano plot showing the fold-change and the *p*-adjust value of gene expression changes comparing RAD *versus* Sham groups. Red: significantly up-regulated expression, blue: significantly down-regulated expression. (**D**) Verification of selected DEGs by qPCR analysis in Sham and RAD mice. Data were presented as fold-change over Sham group (Sham: *n* = 5 or 7; RAD: *n* = 8–9; removed outliers: *Nrarp*-Sham: *n* = 2; *Nrarp*-RAD: *n* = 2). (**E**) Heatmap showing genes related to cell death and survival in the mPFC. Scale bar on the left: standardized rlog-transformed values across samples. (**F**) Gene set enrichment analysis (GSEA) of the transcriptomic changes in the mPFC. The length of the bars indicates the normalized enrichment scores (NES), and the color indicates false discovery rate (FDR). The top enriched gene sets are shown on the right side while the top depleted gene sets are on the left. (**G**) Top panel: Schematic of the experimental procedures. Mice were sacrificed at different time points after cranial irradiation. Bottom panel: Representative images of IBA1-positive microglia in the mPFC. Scale bar, 100 μm. (**H**) Quantification of IBA1-positive microglia in the mPFC (48 h: *n* = 5, 5; 1 wk: *n* = 7, 6; 2 wk: *n* = 8, 9; 4 wk: *n* = 7, 7; 3 m: *n* = 5, 5). (**I**) Representative images of IBA1 (green) and CD68 (red) immunostaining, and three-dimensional (3D) reconstruction (gray) of microglia cells in the mPFC of the Sham and RAD groups. Scale bar, 20 μm. (**J-M**) Quantification of CD68 intensity in IBA1 + area (**J**), IBA1 + cell soma size (**K**), total process length (**L**) and total branch points (**M**). Sham: *n* = 5; RAD: *n* = 7. (**N**) Sholl analysis of microglial morphology in Sham or RAD groups. Data are presented as mean ± s.e.m. and analyzed by multiple *t* tests (**D**, **H**), Student’s *t*-test (**J**-**M**) or two-way ANOVA (**N**)

**Fig. 4:**
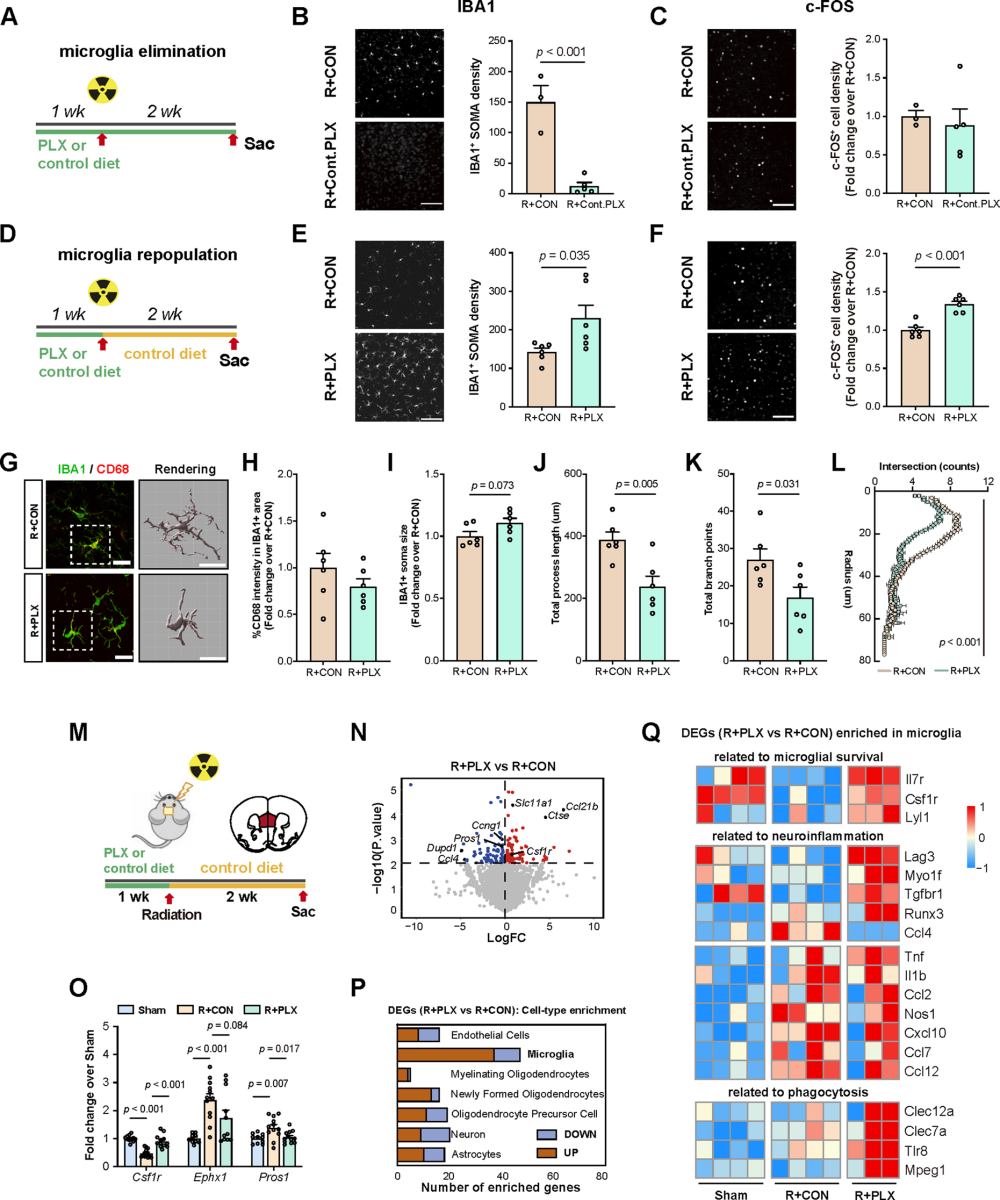
Microglial repopulation restores neuronal activity and drives transcriptomic reprogramming of microglia in the mPFC post irradiation. (**A**) Schematic of the microglia-depletion experiment. Microglia were depleted by continuous administration of PLX3397-containing diet for 3 weeks, started 1 week before 15 Gy cranial irradiation (R + PLX). As a control, mice receiving 15 Gy irradiation were fed with regular chow diet throughout the whole experiment (R + CON). (**B**) Representative images (left panel) and quantification (right panel) of IBA1-positive microglial cell density in the mPFC (R + CON: *n* = 3; R + PLX: *n* = 5). Scale bar: 100 μm. (**C**) Representative images (left panel) and quantification (right panel) of c-FOS-positive cell density in the mPFC (R + CON: *n* = 3; R + PLX: *n* = 5). Scale bar: 100 μm. (**D**) Schematic of the microglial repopulation experiment. Microglia were depleted by continuous administration of PLX3397-containing diet for 1 week before receiving 15 Gy cranial irradiation, and returned to normal chow diet immediately after irradiation for two additional weeks. (**E**) Representative images (left panel) and quantification (right panel) of IBA1-positive microglial cell density in the mPFC (R + CON: *n* = 6; R + PLX: *n* = 6). Scale bar: 100 μm. (**F**) Representative images (left panel) and quantification (right panel) of c-FOS-positive cell density in the mPFC (R + CON: *n* = 6; R + PLX: *n* = 6). Scale bar: 100 μm. (**G**) Representative images of IBA1 (green) and CD68 (red) immunostaining, and three-dimensional (3D) reconstruction (gray) of microglia cells in the mPFC. Scale bar, 20 μm. (**H-K**) Quantification of CD68 intensity in IBA1 + area (**H**), IBA1 + cell soma size (**I**), total process length (**J**) and total branch points (**K**) in the mPFC. R + CON: *n* = 6; R + PLX: *n* = 6. (**L**) Sholl analysis of microglial morphology in the mPFC. (**M**) Schematic of the microglia-repopulation procedures. The mPFC tissues were collected from mice at 2 weeks post irradiation (for male, Sham: *n* = 4; R + CON: *n* = 4; R + PLX: *n* = 3). (**N**) Volcano plot showing the fold-change and the *p*-value of gene expression changes comparing R + PLX mice *versus* R + CON mice. Red: significantly up-regulated expression, blue: significantly down-regulated expression. Only DEGs with *p*-value < 0.01 were presented with color. (**O**) Verification of selected DEGs with qPCR analysis. Data were presented as fold-changes over Sham group (Sham: *n* = 10–11; R + CON: *n* = 12; R + PLX: *n* = 10–12; outliers removed: *Csf1r*-Sham: *n* = 1; *Ephx1-*Sham: *n* = 1; *Pros1-*Sham: *n* = 1; *Cnp*-Sham: *n* = 1; *Csf1r*-R + CON: *n* = 1; *Cnp*-R + PLX: *n* = 1). (**P**) The enrichment analysis of DEGs in specific cell types. (**Q**) Heatmap showing DEGs enriched in microglia related to microglial survival (upper panel), neuroinflammation (middle panel), and phagocytosis (lower panel). Data are presented as mean density ± s.e.m. and analyzed by Student’s t test (**B**, **C**, **E**, **F**, **H**-**K**), two-way ANOVA (**L**), or two way ANOVA with Fisher’s LSD (**O**)

**RNA-seq analysis** All reads were aligned to a mouse reference genome (ftp://ftp.ensembl.org/pub/release-96/fasta/mus_musculus/dna/Mus_musculus.GRCm38.dna.toplevel.fa.gz) using HISAT2 [53]. Uniquely aligned short reads were counted using RSEM [60]. Gene expression was transformed and normalized using TMM normalization using the edgeR package in R. Normalized data was calculated and fitted to negative binomial model with a quasi-likelihood F-test. Differentially expressed genes (DEGs) between the radiation group and the sham group were defined as having an adjusted *p* < 0.1, and DEGs between the groups of radiation with PLX3397 diet and radiation with control diet were defined as having a norminal *p* < 0.01, by Benjamini-Hochberg method in the edgeR. The heatmap and volcano plot of the DEGs were generated with pheatmap and plot packages in R. We used the brain RNA-seq database [111] to assess the cell-type enrichment of the identified DEGs. Using the FPKM numbers in the database, we calculated the cell-type enrichment index using the following formula: FPKM in a given cell type / FPKM in all the other cell types combined. We used the enrichment index of 1.5 as cutoff for considering a transcript as enriched in a specific cell type. We performed the gene-set enrichment analysis (GSEA) to identify KEGG pathways (c2.cp.kegg.v7.2.symbols.gmt) and gene ontology (GO, c5.go.bp.v7.2.symbols.gmt, c5.go.mf.v7.2.symbols.gmt and c5.go.cc.v7.2.symbols.gmt) that were differentially enriched between groups. Gene set size filters were set at minimum of 5 and maximum of 1000. False discovery rates (FDR) for the enrichment score of the gene set were calculated based on 1000 gene set permutations. The gene sets enriched in each group for each category were plotted with ggplot2 (version 3.3.5) in R.

**qPCR** 500 ng RNA from each sample was reverse-transcribed using the QuantiTect Reverse Transcription Kit (Qiagen, Hilden, Germany). 5 ng cDNA was amplified with NovoStart SYBR q-PCR SuperMix Plus (Novoprotein, Shanghai, China) using the following condition: 95 °C for 3 min, followed by 40 cycles of 95 °C for 15 s, 60 °C for 30 s and 72℃ for 20 s. The specificity of the primers was validated by the melting curve analysis. Triplicates of each sample were analyzed, and the average cycle threshold (Ct) value was used in calculating relative expression of target mRNAs using the ΔΔCt method. The relative amount of target mRNA was normalized to the mRNA level of *Gapdh*. The primers used in the qPCR were reported in Supplemental Table S1.

**Minocycline administration** Mice received minocycline administration (Sigma Aldrich, M9511, 30 mg/kg, *i.p.*) for 14 consecutive days after radiation. Control mice received 1×PBS injection.

**Microglial depletion and repopulation** Mice were placed on AIN-76 A rodent diet (Dyets, Bethlehem, PA, USA) containing 290 mg/kg Pexidartinib (PLX3397, MCE, Monmouth Junction, NJ, USA) as described previously [73], for the duration as specified in the text of the result section. Control mice received standard AIN-76 A rodent diet.

**Sorting of microglia** CX3CR1-GFP transgenic mice (The Jackson Laboratory, Stock Number: 005582, Strain Name: B6.129P2(Cg)-Cx3cr1tm1Litt/J) were used here. Mice were randomly subjected to the sham group with regular diet, the radiation group with regular diet, and the radiation with pre-irradiation PLX3397 diet group. Mice were sacrificed and perfused with 10 mL ice-cold 1×PBS at 14 days after whole-brain cranial irradiation. The brain was rapidly removed and transferred into precooled DMEM with 2% FBS (Thermo Scientific, Waltham, MA, USA) and the mPFC tissues were dissected. The mPFC tissues were then digested in preheated DMEM with 2% FBS, 1 mg/mL collagenase VIII, 0.5 mg/mL DNase I (Sigma-Aldrich, St. Louis, MO), 45 µM actinomycin D (Selleck, Houston, TX, USA) for 10 min at 37 °C. Before the digestion was finished, 1 mL DMEM with 10% FBS was added into the buffer to terminate digestion. Samples were filtered through a 70 μm nylon-mesh filter, washed with ice-cold fluorescence-activated cell sorting (FACS) buffer (1×PBS free of calcium and magnesium, 1 mM EDTA, 1% BSA and 3 µM actinomycin D, pH = 7.4), resuspended in 500 µL FACS buffer with DAPI (Sigma, 0.2 ug/mL), and incubated for 5 min at 4 °C. Cells were then washed and resuspended in ice-cold FACS buffer. Cells were sorted by MoFlo Astrios EQs (Beckman Coulter, Indianapolis, IN, USA). Singlets were gated using the height, area and the pulse width of the forward-scatter cells, and cells negative for DAPI were selected as viable cells. The microglia cells were gated as DAPI- GFP + and sorted into a PCR tube.

**LC-MS/MS analysis** For the isolated mouse microglia, the sample preparation was adopted from the micro-scaled proteomic method described by Tsai et al. [100]. Two microliters of lysis buffer that contains 0.2% n-dodecyl-β-D-maltopyranoside (DDM) (D4641, Sigma) in 25 mM ammonium bicarbonate (ABC) (A110537, Aladdin) was added to each sample and then sonicated on ice for 5 cycles (one minute on and off). Next, samples were centrifuged at 3000 × g for 3 min at 4 °C and added with 0.3 µL 100 mM dithiothreitol (ST040, Beyotime). After incubation at 75 °C for 1 h, 0.5 µL 60 mM iodoacetamide (ST1411, Beyotime) were added and incubated at room temperature for 30 min in the dark. Subsequently, 2 µL of 10 ng/µL trypsin (V5280, Promega) in 25 mM ABC was added and incubated at 37 °C for 4 h. Afterward, 2 µL of 10 ng/µL trypsin was added for incubation at 37 °C overnight. Last, 2 µL of 5% formic acid (FA) (F809712, Macklin) was added to quench the reaction. Samples were dried and resuspended with 0.015% DDM in 0.1% FA. The samples were centrifuged at 6000 × g for 1 h at 4 °C, and the supernatant was transferred into a new vial for injection.

Samples were injected to the nano LC 1200 chromatography system (Thermo Scientific) and loaded to a trap column with flow rate 3 µL/min for a volume 15 µL. The peptides were then eluted at a flow rate of 300 nL/min by the LC gradient: 4–8% buffer B (80% acetonitrile) between 0 and 5 min; 8 − 28% buffer B between 5 and 55 min; 28–38% buffer B between 55 and 65 min; 100% buffer B between 65 and 75 min. The peptides were separated and analyzed by DDA (data-dependent acquisition) mode using Orbitrap Fusion Mass spectrometer (Thermo Scientific) with FAIMS Pro™ Interface cycling between CVs of -45 V and − 60 V every 1 s. Mass spectrometry analysis was performed using the following setting: detection mode: positive ion, parent ion scanning range: 350–1500 m/z, dynamic exclusion time 60 s, MS1 resolution of 60 K, AGC target 100% standard, MS1 maximum IT: 50 msec. Peptide secondary mass spectrometry was acquired using the following methods: isolation window: 1.6 Da, activation type: HCD, normalized collision energy: 30, detector type: Ion Trap, AGC Target 1e5, Maximum IT: 35 msec.

**Microglia proteomics data analysis** MS raw data was analyzed with Thermo Scientific Proteome Discoverer 2.4 HT. The Uniprot mouse reference proteome was used as search database. The following database search settings were used: enzyme specificity was full trypsin and maximum two missed cleavage was allowed; precursor mass tolerance was 10 ppm; fixed modifications were cystine carbamidomethylation; variable modifications were methionine (M) oxidation and acetylation of protein N-terminal. Peptide and protein identifications were filtered at 1% FDR. Protein quantification was based on average intensity of top 3 abundant peptides. The protein intensity was log-transformed (base 2) and median-normalized before downstream analysis. Differential protein expression analysis was performed with DEqMS Bioconductor package [114] with a cutoff as *p* < 0.05. Venn plot and heatmaps were generated using VennDiagram (version 1.7.3) and pheatmap (version 1.0.12) packages in R. The function enrichment of differentially expressed proteins (DEPs) was analyzed using ReactomePA (version 1.48.0) package in R/Bioconductor. The top gene sets enriched in each group were plotted with ggplot2 (version 3.3.5) in R.

**Cell death analysis** Mouse-derived microglial cells (BV2 cells) were cultured and used for the cell apoptosis assay. BV2 cells (1 × 10^5^) received 10 Gy irradiation and were cultured for 48 h before analysis. Cells were analyzed using an Annexin V-FITC Apoptosis Detection Kit (BB-4101-1, BestBio) or JC-1 Kit (C2006, Beyotime) following the manufacturer’s instructions. The stained cells were detected using MoFlo Astrios EQs (Beckman Coulter, Indianapolis, IN, USA). For JC-1 staining, the increase in the percentage of JC-1 monomer of total cells indicated mitochondrial depolarization and cell death.

**Enzyme-Linked Immunosorbent Assay** Two weeks after 15 Gy cranial irradiation, the mice were anesthetized for cardiac perfusion with 10 mL 1×PBS. The mPFC tissues were collected, snap-frozen on dry ice and then stored at − 80 °C until analysis. The leukotrience-C4 concentrations were measured with an ELISA kit (cat#EK10010-2, Signalway Antibody, USA) following the manufacturer’s instructions.

**Leukotriene-C4 synthase inhibition** TK05 (cat#HY-117143, MCE, Monmouth Junction, NJ, USA) was used to selectively inhibit LTC4 synthase. Mice received TK05 administration (6 mg/kg, *i.p.*) for 7 consecutive days after radiation.

**Statistical analysis** Detailed information about the sex distribution for all datasets, with female and male mice clearly indicated by red and blue labeling, can be found in Supplementary Table S2. Data were analyzed by GraphPad Prism v.8.02 (San Diego, CA, USA), and presented as the mean ± s.e.m. The Shapiro-Wilk test was conducted to evaluate the normality of the data distribution. Outliers were identified by the Grubbs’ test with an alpha value cutoff as 0.05, and excluded from statistical analysis. Two-tailed unpaired Student’s *t*-test was used to compare two groups, and one-way analysis of variance (ANOVA) or two-way ANOVA followed by *post hoc* tests specified in the figure legends were performed for multiple comparisons. *p* value < 0.05 was considered statistically significant.

## Results

### Decreased mPFC neuronal activities mediate cranial irradiation-induced anxiety-like behaviors and working memory deficits

People exposed to high levels of environmental irradiation or patients who have underwent cranial irradiation treatments have a high incidence of anxiety disorders and memory impairments [51, 68, 69, 89]. Similar to these clinical reports, we observed that compared to the sham-treated group, both sexes of mice received 15 Gy X-ray cranial irradiation exhibited elevated levels of anxiety-like behaviors, including increased grooming in the splash test and less time spent in the illuminated compartment during the light–dark box test at 2 weeks after irradiation, while a trend of reduced total distance travelled in an open field arena was also observed (Fig. 1A -D). Impairment in spatial working memory in the Y-maze test was also observed in the irradiated mice (Fig. 1E).

To identify alteration in brain activity related to cranial irradiation-induced behavioral abnormalities, we collected brains following the completion of behavioral tests and performed immunofluorescence staining of c-FOS, an indicator of neuronal activity [57], across different brain regions that are implicated in emotional regulation and memory processing (Fig. 1F) [28, 39, 85, 86, 101]. We found that irradiation induced an overall pattern of hypoactivation, with significantly decreased c-FOS-positive (c-FOS^+^) cell density in the medial prefrontal cortex (mPFC), anterior cingulate cortex (ACC), lateral bed nucleus of stria terminalis (lBST), insular cortex (IC), basolateral amygdala (BLA), lateral hypothalamus (LH), paraventricular thalamus (PVT), cornu ammonis 3 (CA3) and dentate gyrus (DG) of the dorsal hippocampus (Fig. 1G-H, Figure S1A). Hyperactive amygdala is closely associated with stress-induced anxiety [86]. Surprisingly, we observed a significantly decrease in c-FOS^+^ cell density in the BLA (Fig. 1G), suggesting divergent mechanisms existed in cranial irradiation- and stress-induced anxiety.

We further conducted correlation analyses between the densities of c-FOS^+^ cell and anxiety-related behaviors after irradiation. Among all the brain regions with significantly altered c-FOS expression, changes in the mPFC was significantly and negatively correlated with various anxiety-like behaviors including increased grooming duration in the splash test, decreased time and distance traveled in the center zone of the open field test (Fig. 1I-J, Figure S1B). There was also a tendency of association between mPFC activity and decreased time spent in the illuminated compartment of the light–dark box (Fig. 1K). Notably, cranial irradiation-induced changes in c-FOS^+^ cell density in the mPFC was not due to neuronal loss as indicated by unaltered density of NeuN-labelled neurons, and occurred mainly in the excitatory neurons labelled by CaMK2 (Figure S1 C-G).

To examine the causal relationship between decreased mPFC activity and cranial irradiation-induced behavioral abnormalities, we expressed channelrhodopsin-2 (ChR2) driven by CaMK2α promoter to allow optogenetic activation of mPFC excitatory neurons during behavioral tests (Fig. 2A-B). Optogenetic activation of mPFC excitatory neurons significantly increased c-FOS expression in the mPFC of irradiated mice (Fig. 2C-D). Furthermore, optogenetic activation of mPFC significantly alleviated anxiety-like behaviors as indicated by reduced grooming duration in the splash test, and improved spatial working memory in the Y-maze test, while no significant effect was observed in the open field test (Fig. 2E-G).

Collectively, these data suggested that cranial irradiation induced anxiety-like phenotypes and working memory deficit in mice, accompanied by an overall hypoactive pattern of brain activity. Importantly, our results suggest a critical role of decreased mPFC excitatory neuronal activity in mediating cranial irradiation-induced behavioral abnormality.

### Cranial irradiation induces a delayed yet long-lasting reduction in mPFC microglia density

To explore the potential mechanisms underlying cranial irradiation-induced impaired mPFC activity, we performed whole transcriptomic sequencing (RNA-seq) of mPFC harvested at 2 weeks after 15 Gy cranial irradiation (Fig. 3A)(Supplementary Table S3). A total of 214 differentially expressed genes (DEGs) were identified (*p*-adjusted value < 0.1), and a subset of DEGs was further validated by qPCR (Fig. 3B-D). Notably, among genes that were down-regulated post irradiation, such as *Csf1r*, *Tgfbr1*, and *Egr2*, were known to promote cell survival, proliferation, or homeostasis of microglia [13, 26, 109], whereas genes that were up-regulated, such as *Ephx1*, *Tlr2*, and *Tnfrsf1a*, were known to promote cell death [4, 52, 65] (Fig. 3E). Further gene set enrichment analysis (GSEA) revealed that the top enriched gene sets in the radiation group include “regulation of humoral immune response”, “chemokine activity”, “complement and coagulation cascades”, “regulation of natural killer cell chemotaxis” and “cytokine cytokine receptor interaction”, where are associated with microglia functions [11, 21, 23, 43, 90] (Fig. 3F). Overall, our RNA-seq data suggested that radiation may induce microglia activation and reduction in the mPFC.

To validate these results, we performed immunofluorescence staining of ionized calcium binding adapter molecule 1 (IBA1) to characterize microglia in the mPFC of mice after 15 Gy cranial irradiation. Consistent with our RNA-seq results, we found that the density of IBA1-positive cells in the mPFC was significantly reduced at 2 weeks post irradiation, and this change persisted for at least 3 months, while no significant reduction in microglia density was observed at the 48 h and 1 week time points (Fig. 3G-H), indicating a delayed and persistent effect of 15 Gy irradiation to microglia in the mPFC. Consistently, we found irradiation also induced significant cell death in mouse-derived BV2 microglial cells in vitro (Figure S4 A-D). We further characterized the changes in microglia at 2 weeks post irradiation, a time point at which significant impairments in memory and anxiety-like behaviors were observed. The level of CD68 expression in IBA1-positive cells, a marker of microglial phagocytotic activity, was significantly increased (Fig. 3I-J). Morphological analysis revealed that radiation induced reactive morphology of microglia, as evidenced by increased soma size, significantly decreased process length, and decreased branchpoints of microglia compared to the sham group (Fig. 3K-N). Notably, radiation-induced alterations in microglial density showed regional specificity within the dorsal hippocampus, an area critically involved in memory processing that is also known to exhibit radiation-induced changes in neuronal activity and architecture (Fig. 1G) [77]. We observed a significant decrease in microglial density in the CA1 and DG subregions, as well as in the overlying somatosensory cortex which received radiation exposure (Figure S2). In contrast, no significant alteration in microglia density was obserbed in the CA3 subregion (Figure S2).

### Microglial repopulation, but not suppression of inflammation, alleviates radiation-induced hypoactivation of the mPFC

To examine how the changes in microglia contributed to irradiation induced mPFC hypoactivity, we first suppressed the inflammatory activation of microglia after irradiation through administration of minocycline [55]. Results showed that suppression of microglia activation as indicated by reduced CD68 expression in IBA1-positive microglia, without affecting microglia density, failed to alleviate the reduction in c-FOS^+^ cell density in the mPFC induced by irradiation (Figure S3 A-D). Minocycline treatment also failed to ameliorate radiation-induced anxiety-like behaviors (Figure S3 E-H), suggesting that simply modulating microglial activity may be insufficient to restore mPFC neuronal activity and its associated cognitive outcomes following cranial irradiation.

We next tested whether elimination of microglia could presvent the mPFC hypoactivity induced by irradiation. Chows containing Pexidartinib (PLX3397), a selective ATP-competitive CSF1R and c-KIT inhibitor [26], was supplied to mice to deplete microglia cells throughout the experiment (Fig. 4A). PLX3379 induced significant depletion of microglia, which is similar to reports by other studies [1, 58], but failed to increase c-FOS^+^ cell density in the mPFC and improve anxiety-like behaviors (Fig. 4B-C, Figure S3 I-L). Recent studies have found that reprogramming of microglia by a strategy of depletion followed by repopulation led to neuroprotective effects in a traumatic brain injury mouse model [104]. We next asked whether repopulation of microglia after cranial irradiation could suppress radiation-induced impairments. Towards that end, PLX3379-containing chow was supplied to mice 1 week before irradiation. The mice were returned to normal chow after irradiation. Withdrawal of PLX3397 allowed repopulation of microglia (Fig. 4D-E). Importantly, microglial repopulation led to restoration of mPFC activity, as indicated by the increased density of c-FOS-positive cells (Fig. 4F). Further characterization of the repopulated microglia showed no significant alteration in the level of CD68 within IBA1-labelled microglia after repopulation. Surprisingly, there was a trend towards increased in soma size, and significant reduction in the process length and branchpoints of the repopulated microglia, indicative of enhanced microglia activity (Fig. 4G-L).

To explored the potential molecular profiles of features associated with microglia repopulation in the mPFC after irradiation, we performed RNA-seq analysis of mPFC collected from 15 Gy-irradiated mice with (“R + RLX”) or without (“R + CON”) microglial repopulation. Sham-operated non-irradiated mice (Sham) fed with regular chow were also included (Fig. 4M) (Supplementary Table S4). A total of 183 DEGs (Deseq2, *p*-value < 0.01) were identified when comparing “R + PLX” with “R + CON” group, with a subset of DEGs validated by qPCR (Fig. 4N-O). Cell-type specificity analysis of the DEGs showed that a majority of DEGs associated with microglial repopulation in the irradiated mPFC were microglia-enriched (Fig. 4P). Among these 47 DEGs, genes functionally involved in the proliferation and survival of microglia and myeloid cells (*Il7r*, *Csf1r*, *Lyl1*,) [26, 79, 96], suppression of neuroinflammation (*Lag3*, *Myo1f*, *Tgfbr1*, *Runx3*, ) [47, 63, 66, 97], and promotion of phagocytosis (*Clec12a*, *Clec7a*, *Tlr8*, *Mpeg1*) {Gambuzza, 2014 #87;Jiao, 2022 #88} [18] were upregulated, while pro-inflammatory mediator *Ccl4* [16]was down-regulated (Fig. 4Q). Notably, we also observed upregulation of inflammation-associated cytokines and chemokines (*Tnf*, *Il1b*, *Ccl2*, *Nos1*, *Cxcl10*, *Ccl7*, *Ccl12*) in the mPFC of irradiated mice, which have been reported previously in microglial transcriptome in response to irradiation [75]. However, expression of these genes was not altered by PLX-induced microglia regeneration, arguing the pathological roles of these factors in regulating neuronal activity and cognitive functions in radiation-induced brain injury as previously considered (Fig. 4Q).

### Alterations in the proteomic profiles of mPFC microglia induced by cranial irradiation and microglia repopulation

**Fig. 5:**
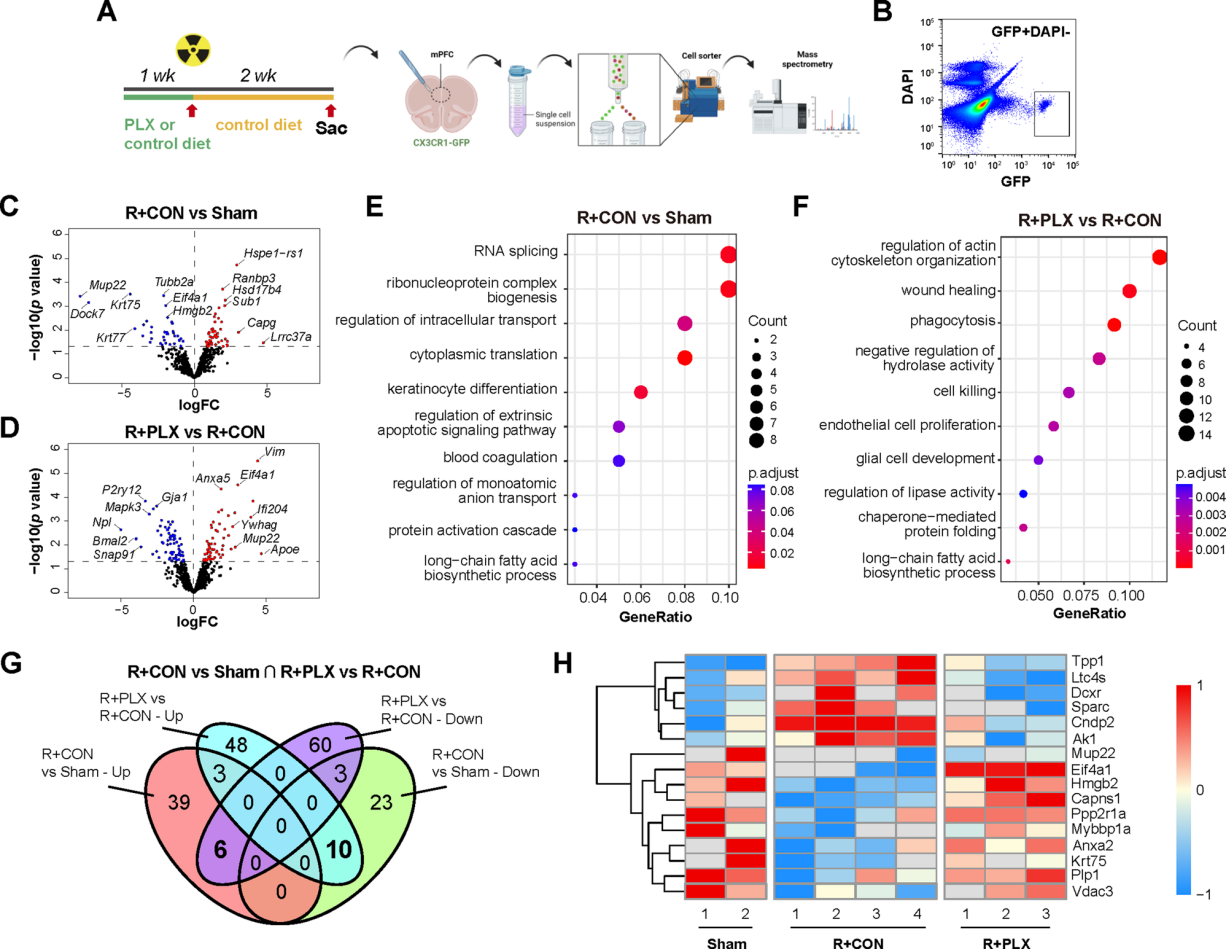
Proteomic profiling reveals radiation-induced leukotriene biosynthesis in the mPFC is reversed by microglial repopulation. (**A**) Schematic of the microglial repopulation, mPFC tissue collection, microglia cell sorting and mass spectrometry-based proteomics analysis (for male, Sham: *n* = 2; R + CON: *n* = 4; R + PLX: *n* = 3). (**B**) Gating strategy and representative plots of viable microglia (GFP-positive and DAPI-negative) in the mPFC. (**C-D**) Volcano plots of the differentially expressed proteins (DEPs) comparing the “R + CON” with “Sham” groups (C) and the “R + PLX” with “R + CON” groups (D). Blue and red dots represent significantly down-regulated and up-regulated DEPs, respectively. (**E-F**) Plots showed top enriched pathways of DEPs comparing the “R + CON” with “Sham” groups (E), and the “R + PLX” with “R + CON” groups (F), revealed by gene ontology analysis. (**G**) Venn plot of the overlap DEPs between groups. Bold-labeled numbers represented DEPs with opposite directions of changes in different groups. (**H**) Heatmap showing the DEPs with opposite directions of changes among three groups in the microglia sorting from the mPFC. Scale bar on the left. Sham: non-irradiated mice without microglial repopulation, R + CON: mice received 15 Gy irradiation without microglial repopulation, R + PLX: mice received 15 Gy irradiation with microglial repopulation

Given that microglia repopulation restored mPFC neuronal activity after irradiation, we next investigated how irradiation disrupted microglial function and the mechanisms by which regenerated microglia facilitated functional recovery of mPFC neurons. To this end, we sorted 1000 viable microglia (GFP^+^DAPI^−^) from the mPFC of CX3CR1-GFP mice by flow cytometry. Mass spectrometry-based micro-scaled proteomics sequencing were performed on the same amount of microglia collected from each group (Fig. 5A-B) (Supplementary Table S5). A total of 84 differentially expressed proteins (DEPs) were identified (DEqMS, *p* < 0.05) in the mPFC microglia when comparing the “R + CON” group with the “Sham” group (Fig. 5C). Gene Ontology analysis identified significant enrichment in biological processes including RNA splicing, regulation of extrinsic apoptotic signaling pathway and protein activation cascade (Fig. 5E), suggesting apoptosis and abnormal activation of microglia induced by irradiation. When comparing the “R + PLX” group with the “R + CON” group, 130 DEPs were identified (Fig. 5D), and were functionally enriched for regulation of actin cytoskeleton organization, wound healing, endothelial cell proliferation and glial cell development (Fig. 5F), which stand for the potential mechanisms underlying restored activity of the mPFC after repopulation of microglia cells. Further comparison of the identified DEPs revealed 22 overlapped DEPs between different groups (Fig. 5G). Among these DEPs, glycolysis-enhancing factors (AK1, DCXR) [29, 49] and pro-inflammatory factors (LTC4S, SPARC) [80, 87] were found increased in “R + CON” group but not “R + PLX” group when compared to “Sham” group (Fig. 5H). Proteins that are known to regulate cell proliferation (EIF4A1, CAPNS1, MYBBP1A) [24, 72, 113], suppress inflammatory response (PPP2R1A, PLP1) [9, 37], or suppress ROS production and cell death (VDAC3) [84] were found reduced in “R + CON” group but not in “R + PLX” group (Fig. 5H). These results suggest that radiation induced increased glycolysis, inflammatory response and leukotriene biosynthesis, while suppressing cell proliferation of microglia, indicating a possible involvement of glycolysis-induced activation and inflammatory responses of microglia in radiation-induced impairment of mPFC activity and cognitive functions.

### Suppress LTC4 production reverses radiation-induced anxiety-like phenotype and memory deficits

**Fig. 6:**
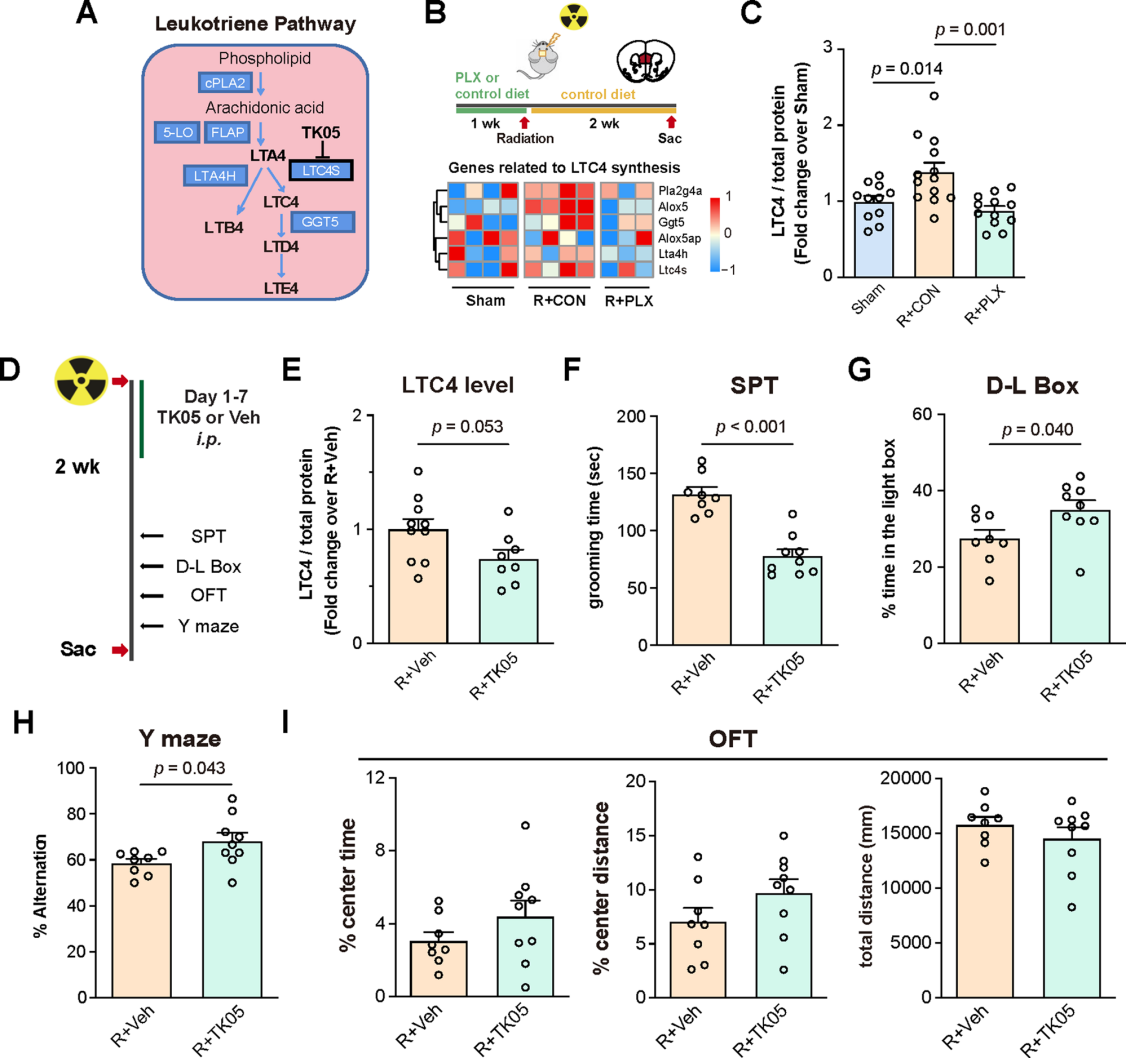
Suppression of LTC4S-driven leukotriene C4 synthesis reverses radiation-induced anxiety and memory deficits. (**A**) Diagram of leukotriene synthesis pathway. (**B**) Upper panel: schematic of microglial repopulation and cranial irradiation procedures. Lower panel: heatmap of RNA-seq results showing the expression levels of genes related to leukotriene-C4 (LTC4) synthesis in the mPFC. (**C**) Quantification of LTC4 levels in the mPFC between groups. Data were presented as fold-change over Sham group (Sham: *n* = 11; R + CON: *n* = 13; R + PLX: *n* = 12; 1 outlier excluded from R + PLX group). (**D**) Schematic of the experimental designs. Following cranial irradiation, mice were administrated with TK05 (6 mg/kg, *i.p.*) for 1 week (R + TK05), followed by the behavioral tests conducted 2 weeks post irradiation. As a control, mice were administrated with saline for 1 week post irradiation (R + Veh). (**E**) Quantification of LTC4 levels in the mPFC between groups. Data were presented as fold-change over R + Veh group (R + Veh: *n* = 10; R + TK05: *n* = 8; 1 outlier excluded from R + TK05 group). (**F**) Quantification of the grooming duration in the splash test. (**G**) Quantification of time spent in the light box as a percentage of the total time in the D-L Box. (**H**) Quantification of the % Alteration in the Y maze test. (**I**) Quantification of the time spent in the center zone (as percentage of total time), the center distance travelled (as percentage of total distance travelled), and total distance travelled in the open field test. (**F**-**I**) R + Veh: *n* = 8; R + TK05: *n* = 9. Similar amount of male and female mice was used for each group and the results were combined for statistical analysis. Data are presented as mean ± s.e.m. and analyzed by one-way ANOVA with *Tukey* post hoc test (**C**), or Student’s *t* test (**E**-**I**)

LTC4S catalyzes leukotriene-C4 synthesis, a potent immune-regulating lipid mediator derived from arachidonic acid metabolism (Fig. 6A) [41]. Our proteomic analysis revealed that cranial irradiation increased LTC4S levels in microglia of the mPFC, an effect that can be alleviated through microglia replenishment. Associated with thses findings, our bulk RNA-seq data from the mPFC demonstrated similar alterations in the expression of genes involved in the leukotriene C4 biosynthetic pathway, including *Pla2g4a* (encoding cPLA2), *Alox5* (encoding 5-LO), *Ggt5*, *Lta4h* and *Ltc4s* (Fig. 6B). Furthermore, we confirmed the increased LTC4 levels in the mPFC following irradiation, which was also reversed by microglia replenishment (Fig. 6C). Similar observations were found in the irradiated BV2 microglial cells, which showed elevated LTC4S protein expression and LTC4 synthesis (Figure S4 E-G).

To further investigate the role of LTC4 in irradiation-induced behavioral abnormality, mice were administrated with TK05, a potent and selective inhibitor of LTC4S [54] after 15 Gy irradiation for 1 week (Fig. 6D). The results showed that TK05 significantly reduced LTC4 level in the mPFC compared to control mice received vehicle administration (Fig. 6E). Compared to the vehicle-treated group, administration of TK05 suppressed anxiety-like behaviors, including reduction of overgrooming in the splash test and increased time spent in the light box in the dark-light box test (Fig. 6F-G). TK05 administration also attenuated impairment in spatial working memory test in the Y maze, while no significant effect was observed in the open field test (Fig. 6H-I).

Taken together, our data suggested increased LTC4S expression in the mPFC microglia as a key mechanism underlying irradiation-induced behavioral abnormaltiy. Specifically, targeting LTC4S, rather suppressing microglia activation, might support the treatment of irradiation-induced cognitive dysfunctions.

## Discussion

In this study, we demonstrated that cranial irradiation reduced neuronal activity in the mPFC, which was functionally associated with anxiety-like behaviors and memory deficits. Despite changes in radiation-induced inflammatory state and proteomic profiles of microglia within the mPFC were observed, we found that microglia replenishment, rather than consistent depletion or minocycline treatment, effectively restored mPFC neuronal activity post irradiation. Furthermore, we identified elevated leukotriene-C4 synthesis as a major contributor to radiation-induced anxiety-like behavior and memory decline in mice. These findings suggest that leukotriene C4 produced by microglia, rather than pro-inflammatory cytokines and chemokines, is the primary driver of cognitive impairments and mood disorder in the early stage of radiation-induced brain injury.

Our study revealed that mPFC activity was significantly reduced after radiation, and c-FOS expression in the mPFC was significantly and negatively correlated with anxiety-like behaviors post irradiation. We further showed that optogenetic activation of the mPFC can prevent radiation-induced anxiety-like phenotypes and memory impairment, demonstrating the casual role of mPFC activity in regulating radiation-induced behavioral deficits. The mPFC has reciprocal connections with many brain regions within the limbic system that regulate emotion and memory [34, 108] and is thought to be a hub that integrates external and internal information to evaluate the safety of an environment and govern the decision to switch between exploratory and defensive/avoidance behaviors [39, 91, 92]. The mPFC is susceptible to radiation-induced injury [6, 59, 82]. Consistently, magnetic resonance imaging studies of patients with radiation-induced brain injury showed that both the frontal cortex and hippocampus were particularly susceptible to radiation-induced atrophy [15, 17, 82]. Our results suggest that the impairment of mPFC function by radiation may weaken animals’ ability to make informed decisions, which, in turn, impair memory performance in Y-maze or generate anxiety-like behaviors, which can be functionally rescued by optogenetically increasing the excitatory neuronal activity of the mPFC.

To investigate the cellular and molecular mechanisms of radiation-induced hypoactivity of the mPFC, we performed RNA-seq analysis, which revealed two interesting features: (1) enrichment of DEGs involved in immune processes and signaling pathways that are commonly mediated by microglia and (2) changes in DEGs that promote microglial depletion. Elevated microglial pro-inflammatory response, increased cellular senescence, and reduced cell density are pathological features that have been previously reported in brains following cranial irradiation [42, 46, 102]. However, the molecular signatures of microglia and their associated factors involved in the progression of radiation-induced behavioral disorders remain largely unexplored. Our RNA-seq analysis of the mPFC revealed a reduction of microglial survival genes, including *Csf1r*,* Tgfbr1*, and *Egr2*, along with an increase in apoptosis and anti-proliferative genes, such as *Ephx1* and *Tnfrsf1a* [52, 65, 73, 109]. These findings elucidate the molecular profiles associated with radiation-induced microglial loss, highlighting changes in crucial genes that potentially regulate microglial survival and proliferation, thereby contributing to the functional alterations of mPFC neurons post irradiaiton.

A recent report using single-cell RNA-sequencing revealed that radiation induced complex microglial responses in the hippocampus of juvenile mice, including up-regulation of pro-inflammatory cytokines and chemokines such as *Tnf*, *Il1b*, *Il12b*, and *Ccl2*, and down-regulation of anti-inflammation genes such as *Scl4a7*, *Sepp1*, *Mrc1* and *Tlr8* [75]. Minocycline, which inhibits microglial activation and neuroinflammation, has also been reported to attenuate the production of pro-inflammatory factors in mice challenged with LPS [44] or receiving whole body irradiation [70]. However, our study found that systemic minocycline administration failed to prevent radiation-induced detrimental effects in neuronal acitivity and microglia density in the mPFC. In line with our findings, clinical studies showed that anti-inflammatory treatments with minocycline do not mitigate memory deficits and other symptom burdens of irradiation treatment such as fatigue and interferes with activities of daily life [20, 40]. Futher, corticosteroid-based treatments show effective anti-inflammatory efficacy as therapeutic agents for infectious diseases of the brains and in mouse model of multiple sclerosis, significantly down-regulating pro-inflammatory cytokines such as IL-1β, IL-6, IL-17, IFN-γ, and TNF-α [31, 83]. However, only a subset of patients with radiation-induced brain injury respond to corticosteroid treatment [106]. It is also worth noting that in our study, while microglial replenishment led to the restoration of neuronal activity in the mPFC in irradiated mice, the transcriptomic profiles showed no alterations on the expression of pro-inflammatory cytokines and chemokines, including *Tnf*, *Il1b*, *Nos1*, *Ccl2*, *Cxcl10*, *Ccl7*, and *Ccl12*. These findings suggest that distinct mechanisms other than pro-inflammatory cytokines may contribute to radiation-induced pathological processes [19, 106], calling for a revisit of the pathological roles of pro-inflammatory factors in radiation-induced neuronal hypoactivity and cognitive impairment in the early stage (two weeks post irradiation) of radiation-induced brain injury.

Emerging evidences suggest that microglial replenishment may prevent brian injury or facilitate brain repair in mouse models of neurodegenerative diseases [22, 26, 45, 110]. As for radiation-induced memory impairments, pre- or post irradiation treatment of PLX5622 for microglial elimination followed by microglial regeneration has previously been shown to prevent mice from working memory deficits, protect from loss of dendritic spines in the hippocamus, and rescue long-term working memory impairment [30, 58]. While these studies suggested that microglia replenishment protected mice from hippocampus-dependent memory deficits, the detailed mechanisms on how microglial state affects the pathological changes in mice that receive cranial irradiation remain unaddressed.

Our study showed that instead of continuous elimination, replenishment of microglia produced beneficial effects in reserving neuronal activity of the mPFC in mice that received single dose of 15 Gy cranial irradiation. To gain functional insights into the therapeutic mechanism of microglial replenishment, we conducted a proteomic analysis, sorted microglia from the mPFC, and identified 22 DEPs whose expression patterns were significantly altered by radiation but reversed upon microglia replenishment. Notably, radiation induced upregulation of AK1 and DCXR, regulatory factors known to enhance glycolysis in microglia and tumor cells, an effect that was mitigated following microglia replenishment [29, 49]. AK1 catalyzes the conversion of ATP to ADP, decreasing the ATP/ADP ratio and promoting aerobic glycolysis, a metabolic hallmark of inflammatory microglia [29, 88, 107]. Similarly, DCXR involves in xylulose metabolism and plays a crucial role in maintaining glycolytic capacity and cancer cell proliferation [49]. These findings suggest that microglial replenishment reverses radiation-induced metabolic alterations associated with neuroinflammation.

Furthermore, microglial proteins that were found to be upregulated by radiation but suppressed in replenished microglia included LTC4S, an enzyme required for the synthesis of leukotriene C4 that involves in inflammatory responses, and SPARC, a secreted protein that mediates cell-extracellular matrix interaction and inflammatory responses [56, 74]. Concurrently, replenished microglia showed increased protein levels of PPP2R1A and PLP1. PPP2R1A is a regulatory subunit of protein phosphatase 2, which negatively regulates angiotension II- or PMA-induced reactive oxygen species (ROS) production and inflammatory response in microglia [9]. PLP1 is a key component of myelin, which is essential for maintaining myelin stability and preventing excessive microglial activation and neurinflammation [5, 38]. Changes in these proteins indicate a functional shift of replenished microglia towards an anti-inflammatory state in the irradiated mPFC. Additionally, increased protein levels of EIF4A1, CAPNS1, MYBBP1A, and VADC3 were also found in the replenished microglia. EIF4A1 is an ATP-dependent RNA helicase, which supports efficient protein translation during cell cycle progression [24]. CAPNS1 is the small subunit of calpain, which regulates phagocytic activity and microglial proliferation [113]. The downregulation of MYBBP1A and loss of VDAC3 function are associated with increased cell death rate [72, 84]. Changes in these proteins also align with the restored cell density observed in replenished microglia in the mPFC following radiation.

Leukotrienes are eicosanoids derived from arachidonic acid metabolism. Among them, leukotriene C4 exhibits high biological activity as an immune mediator, interacting with CysLTR1 and CysLTR2 receptors to elicit peripheral immune allergic responses [81]. LTC4S functions as a membrane GSH S-transferase, catalyzing the conjugation of leukotriene A4 with glutathione to synthesize LTC4, which is predominantly expressed in peripheral immune cells such as eosinophils, mast cells, monocytes, and microglia in the CNS [71]. Inflammatory stimuli, like lipopolysaccharide, are known to elevate LTC4S expression and LTC4 synthesis, along with increased COX-2 and prostaglandin E2 production, thereby participating in inflammatory process [32, 94]. Studies have demonstrated that LTC4 and its receptor CysLTR1 play pivotal roles in endoplasmic reticulum stress and the generation of ROS by clinical chemotherapy drugs such as doxorubicin and 5-FU, leading to DNA damage and cell death [25]. Furthermore, CysLTR1 mediates calcium accumulation in monocytes and macrophages, subsequently activating immune signaling pathways including p38, ERK1/2, and NF-κb [105, 112]. LTC4 has also been implicated in promoting blood-brain barrier (BBB) disruption and inducing cerebral edema, and the use of CysLTR2 inhibitor HAMI3379 or specific knockdown of CysLTR2 expression significantly reduces post-ischemic reperfusion induced brain necrosis [14, 93]. However, the potential pathological role of microglia-secreted LTC4 to the dysfunctions of memory and mood stability in the irradiated mice remains unexplored. Our study revealed that LTC4S protein, a key enzyme for leukotriene C4 biosynthesis, and LTC4 levels were both upregulated in radiation-exposed microglia and the mPFC post irradiation. Our results demonstrated that by direct supressing radiation-induced LTC4 synthesis with an LTC4S inhibitor, a significant improvement in working memory performance and alleviation of radiation-induced anxiety-like behaviors were observed in mice that received crainial irradiaiton. These findings suggest that strategies for blocking LTC4 synthesis or pharmacological inhibition of LTC4 receptors may carry therapeutic potential for radiation-induced brain dysfunction and mood disorders.

While microglial-derived LTC4 appears to drive radiation-induced cognitive deficits in mice, yet depletion of microglia fails to show protective effects. Our observation reiterates the dual nature of microglial functions, in which while microglia may produce pathological factor (like LTC4) to impair neuronal function, microglia also provide essential neuroprotective functions. Indeed, it has been shown that microglia maintain neuronal network activity during excitotoxic injury in stroke mouse model and facilitate clearance of damaged cellular components such as myelin debris [7, 8, 98]. In our radiation-induced brain injury model, continuous microglia depletion may eliminate detrimental LTC4 production while simultaneously depriving the homeostatic functions provided by microglia, potentially explaining the lack of behavioral improvement.

It is worth noting that previous clinical and preclinical studies have demonstrated sex-specific responses to radiation exposure. Reports from pediatric brain tumor patients and mouse models have shown varying degrees of cognitive impairment between sexes [10, 78]. In our study, we maintained balanced groups of male and female mice for most experiments and observed comparable radiation-induced effects across both sexes. The absence of significant sex differences in our current findings may be attributed to several factors. For example, our study employed a distinct radiation paradigm, a single 15 Gy dose delivered via X-ray to the mouse head, whereas other studies reporting sex differences used different type of radiation exposure (charged helium particles) and delivery method (whole-body irradiation) at substantially lower doses (5 ~ 30 cGy) [78]. Additionally, the timing of behavioral assessment needs to be considered, as we evaluated cognitive function two weeks post irradiation, while other studies demonstrating sex differences conducted testing at later time points (12 weeks post irradiation), when compensatory mechanisms may merge differentially between sexes.

Our results also demonstrated that continuous PLX3397-mediated microglia depletion following 15 Gy cranial irradiation failed to improve anxiety-like behaviors. This finding contrasts with the beneficial effects reported previously [1], which used a different experimental paradigm (9 Gy irradiation in older mice assessed 4–6 weeks post irradiation). Several differences may account for these divergent outcomes, including radiation dose (15 Gy vs. 9 Gy), animal age (2–3 months vs. 6 months), post irradiation assessment time of behaviors (2 weeks vs. 4–6 weeks), behavioral tasks used (anxiety-like behaviors/Y-maze vs. object recognition/fear conditioning), and different CSF1R inhibitor used (PLX3397 vs. PLX5622). These variables highlight the importance of context when evaluating microglial modulation strategies and their outcomes. This interpretation aligns with our minocycline findings, where reduced microglial activation by minocycline treatment similarly failed to improve radiation-induced neuronal hypoactivity and cognitive impairment. Together, these results suggest that selective modulation of specific microglial pathways, rather than global depletion or suppression, may represent a more promising therapeutic approach.

## Conclusions

In summary, our study reveals that radiation-induced hypoactivation of the mPFC plays a crucial role in anxiety-like behaviors and memory deficits, with alterations in microglial leukotriene C4 metabolism serving as a key mediator. These results highlight the specific inhibition of LTC4 synthesis, rather than general suppression of microglial activation and pro-inflammatory cytokine expression, as a promising therapeutic strategy for alleviating radiation-induced cognitive and mood disorders.

## Electronic supplementary material

Below is the link to the electronic supplementary material.

Supplementary Material 1

## Data Availability

Detailed information about the sex distribution for all datasets, with female and male mice clearly indicated by red and blue labeling, can be found in Supplementary Table S2. RNA-seq datasets of mPFC can be found in Supplementary Table S3 and Table S4. Proteomic datasets of mPFC microglia can be found in Supplementary Table S5. Other data from the current study are available upon reasonable request from the lead contact, Wei-Jye Lin (linwj26@mail.sysu.edu.cn).

## Abbreviations

AAV: Adeno-associated viruses
ACC: Anterior cingulate cortex
ANOVA: Analysis of variance
BLA: Basolateral amygdala
IC: Insular cortex
CA3: Cornu ammonis 3
ChR2: Channelrhodopsin-2
CSF1R: Colony stimulating factor 1 receptor
Ct: Cycle threshold
DEGs: Differentially expressed genes
DEPs: Differentially expressed proteins
DG: Dentate gyrus
FACS: Fluorescence-activated cell sorting
FDR: False discovery rates
GSEA: Gene-set enrichment analysis
IBA1: Ionized calcium binding adapter molecule 1
IBST: Lateral bed nucleus of stria terminalis
LH: Lateral hypothalamus
LTC4: Leukotriene C4
mPFC: Medial prefrontal cortex
PVT: Paraventricular thalamus
RIBI: Radiation-induced brain injury
RIN: RNA integrity number
ROS: Reactive oxygen species
