# Supplementary material for: Microglial repopulation reverses radiation-induced cognitive dysfunction by restoring medial prefrontal cortex activity and modulating leukotriene-C4 synthesis
Source: Acta Neuropathol Commun. 2025 May 19;13:105. doi: 10.1186/s40478-025-02026-8 (PMC12087111; doi:10.1186/s40478-025-02026-8)
Supplement: Supplementary file 1 — Supplementary Material 1 [file 40478_2025_2026_MOESM1_ESM.pdf]

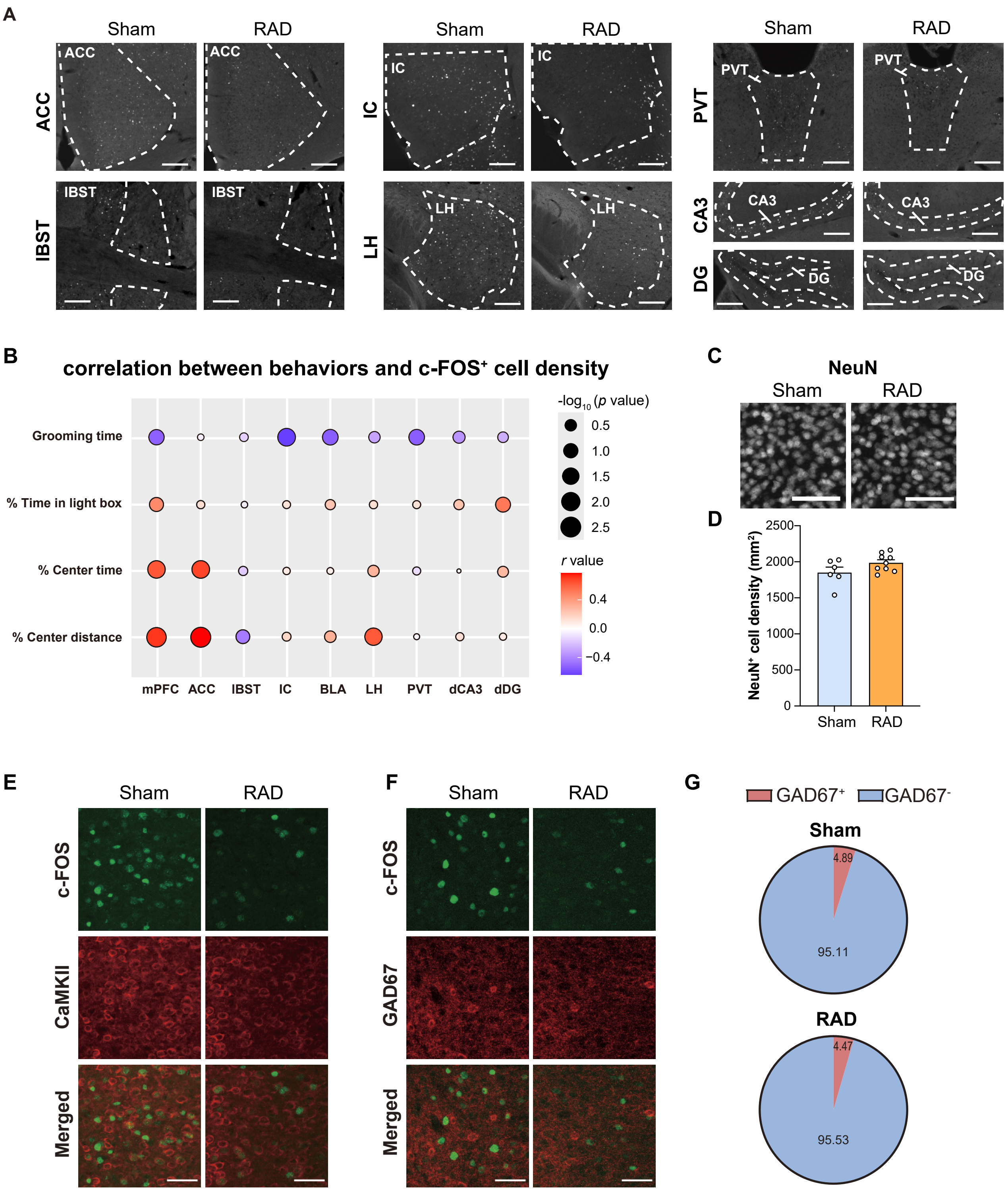

A
Sham
RAD
ACC
IC
PVT
IBST
LH
CA3
DG
B
correlation between behaviors and c-FOS+ cell density
Grooming time
% Time in light box
% Center time
% Center distance
mPFC ACC IBST IC BLA LH PVT dCA3 dDG
-log10 (p value)
0.5
1.0
1.5
2.0
2.5
r value
0.4
0.0
−0.4
C
NeuN
D
NeuN+ cell density (mm2)
2500
2000
1500
1000
500
0
E
c-FOS
CaMKII
Merged
F
GAD67
G
GAD67+ GAD67-
4.89
95.11
4.47
95.53

Supplementary Figure 1. Cranial irradiation-induced neuronal hypoactivity in the mPFC correlates to anxiety-like behaviors and is independent of neuronal loss. (**A**) Representative images of c-FOS immunostaining in the anterior cingulate cortex (ACC), lateral bed nucleus of stria terminalis (lBST), insular cortex (IC), lateral hypothalamus (LH), paraventricular thalamus (PVT), cornu ammonis 3 (CA3) and dentate gyrus (DG) of the dorsal hippocampus. Sham: mice without crainial irradiation, RAD: mice received cranial irradiation for two weeks. Scale bar: 200μm. (**B**) Correlation of anxiety-like behavioral indexes with the density of c-FOS positive cells in different brain regions with significantly altered neuronal activity. (**C-D**) Representative images of NeuN-positive cells (C), and quantification of NeuN-positive neurons in the mPFC. Sham: n=6; RAD: n=9. Data were represented as mean ± s.e.m. and analyzed by Student's *t* test. Scale bar: 100 μm. (**E**) Representative images of co-localization of c-FOS-positive (green) and CaMKII-positive excitatory neurons (red) in the mPFC of Sham and RAD groups. Scale bar: 50 μm. (**F**) Representative images of co-localization of c-FOS-positive (green) and GAD67-positive inhibitory neurons (red) in the mPFC of Sham and RAD groups. Scale bar: 50 μm. (**G**) Percentage of c-FOS-positive cells which were also GAD67-negative ($GAD67^-$) or GAD67-positive ($GAD67^+$ cells in the mPFC. Data are presented as mean percentage of c-FOS-positive cells.

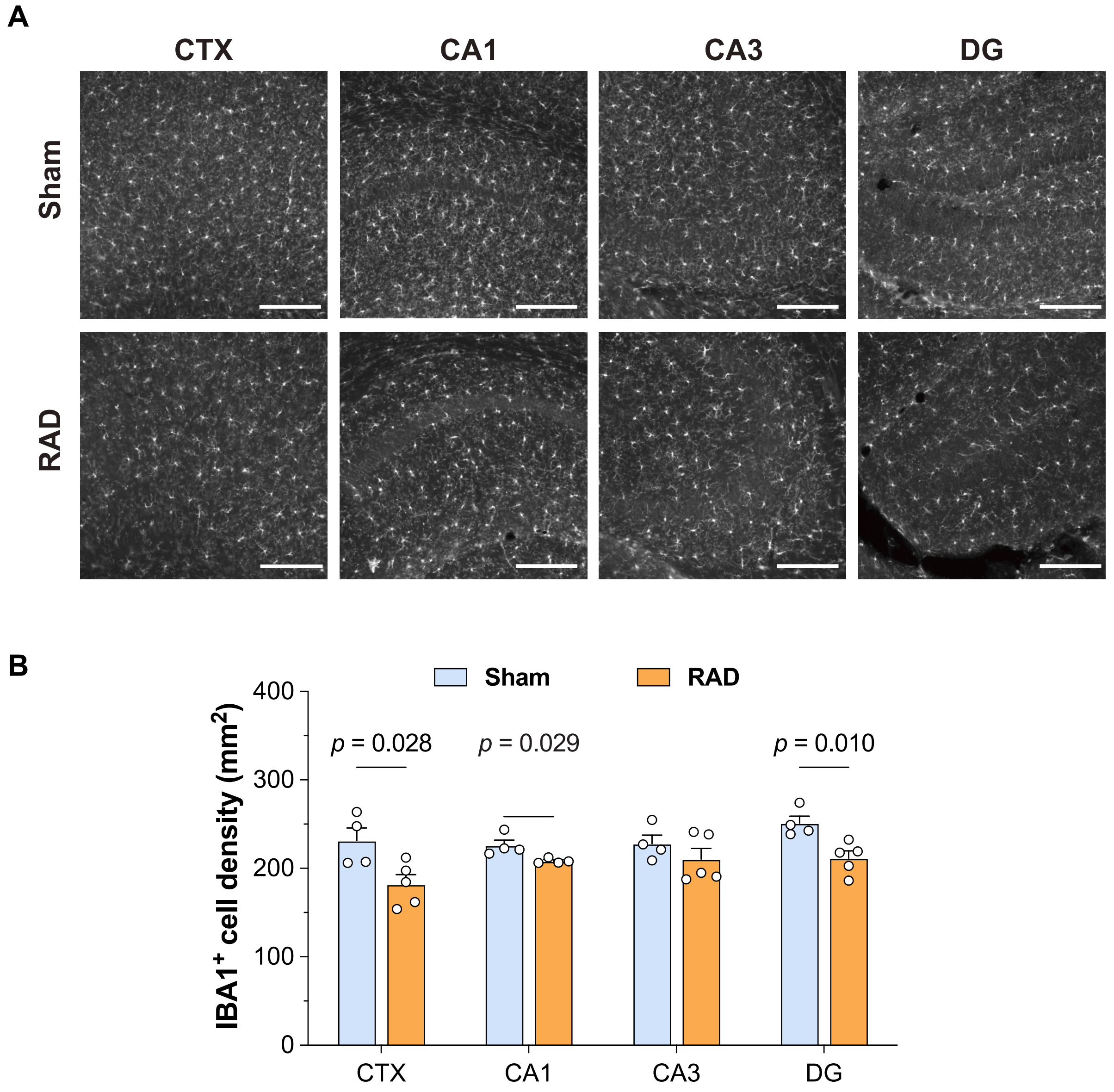

Supplementary Figure 2. Cranial irradiation reduces microglial cell density in the dorsal hippocampus and somatosensory cortex. (**A**) Representative images of IBA1-positive cells in the somatosensory cortex (CTX), cornu ammonis 1 (CA1), cornu ammonis 3 (CA3) and dentate gyrus (DG) of the dorsal hippocampus in the Sham and RAD groups. Scale bar: 200µm. (**B**) Quantification of the IBA1-positive cell density in the somatosensory cortex and hippocampal subregions (Sham: n= 4; RAD: n= 4-5). Data are presented as mean ± s.e.m. and analyzed by multiple *t* tests.

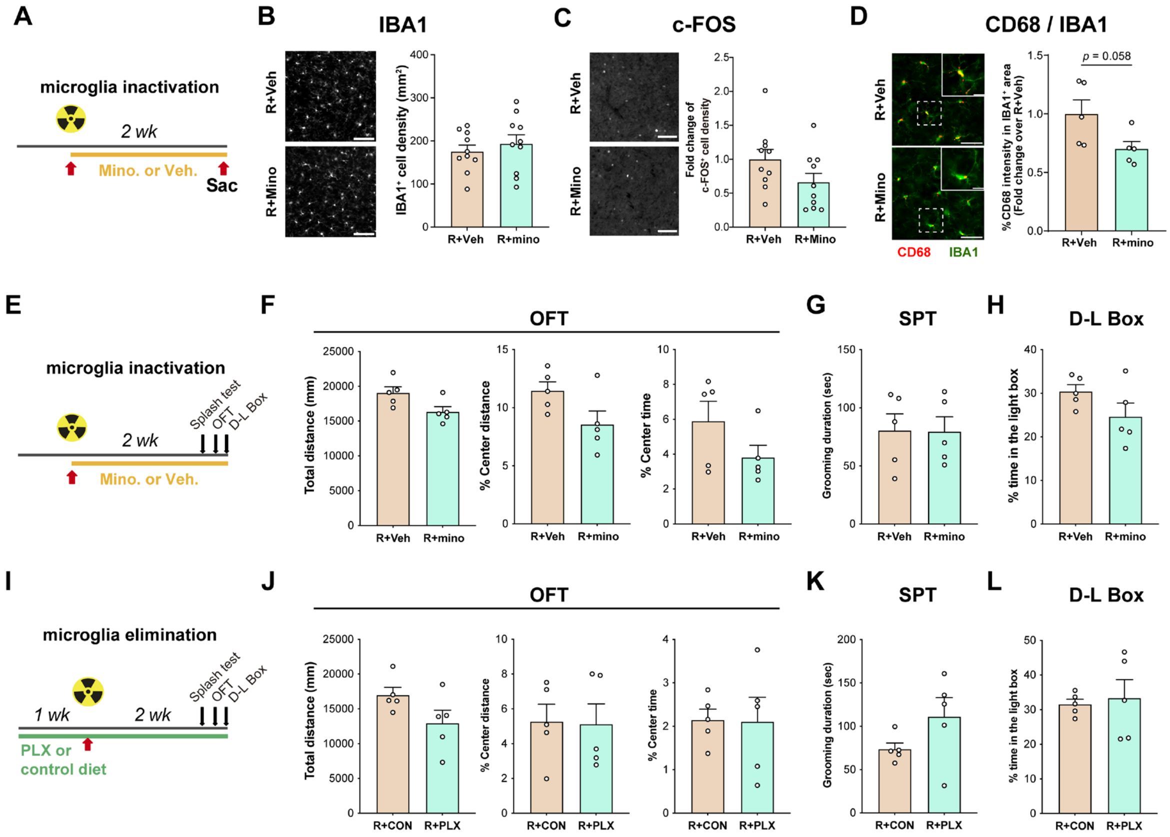

Supplementary Figure 3. Minocycline treatment or continuous microglial depletion fails to ameliorate radiation-induced microglial loss, neuronal hypoactivity in the mPFC, and behavioral deficits. (**A**) Schematic of the experimental designs. Minocycline (R+mino, 30mg/kg, *i.p.*) or vehicle (R+Veh) was continuously administered post-irradiation. Tissue harvest (Sac) was conducted at 2 weeks (2 wk) post irradiation. (**B**) Representative images (left panel) and quantification (right panel) of IBA1-positive microglial cell density in the mPFC. Scale bar: 100μm. (**C**) Representative images (left panel) and quantification (right panel) of c-FOS-positive cell density in the mPFC. Scale bar: 100μm. (**D**) Representative images of CD68 (red) and IBA1 (green) immunostaining

and quantification of CD68 intensity in IBA1+ area of microglia cells in the mPFC. Scale bar, large image: 40 µm, small image: 10 µm. (**E**) Schematic of the experimental designs. Minocycline (R+mino) or vehicle (R+Veh) was continuously administered post-irradiation. Behavioral tests were conducted at 2 weeks (2 wk) post irradiation. (**F, J**) Quantification of total distance travelled, percentage of the time spent in center zone, and travelled distance in center zone as a percentage of the total distance in the open field test. (**G, K**) Quantification of the grooming duration during the splash test. (**H, L**) Quantification of time spend in the light box as a percentage of the total time in the D-L Box. (**B**-**C**): R+Veh: n=10; R+mino: n=10. (**D, F**-**H**): R+Veh: n=5; R+mino: n=5. (**I**) Schematic of the microglia-depletion experiment. Microglia were depleted by continuous administration of PLX3397-containing diet for 3 weeks, started 1 week before 15 Gy cranial irradiation (R+PLX). As a control, mice receiving 15 Gy irradiation were fed with regular chow diet throughout the whole experiment (R+CON). Behavioral tests and tissue harvest (Sac) were conducted at 2 weeks (2 wk) post irradiation. (**J**-**L**): R+CON: n=5; R+PLX: n=5. Data are represented as mean ± s.e.m. and analyzed by student's *t* test (**B**-**D**, **F**-**H**, **J**-**L**).

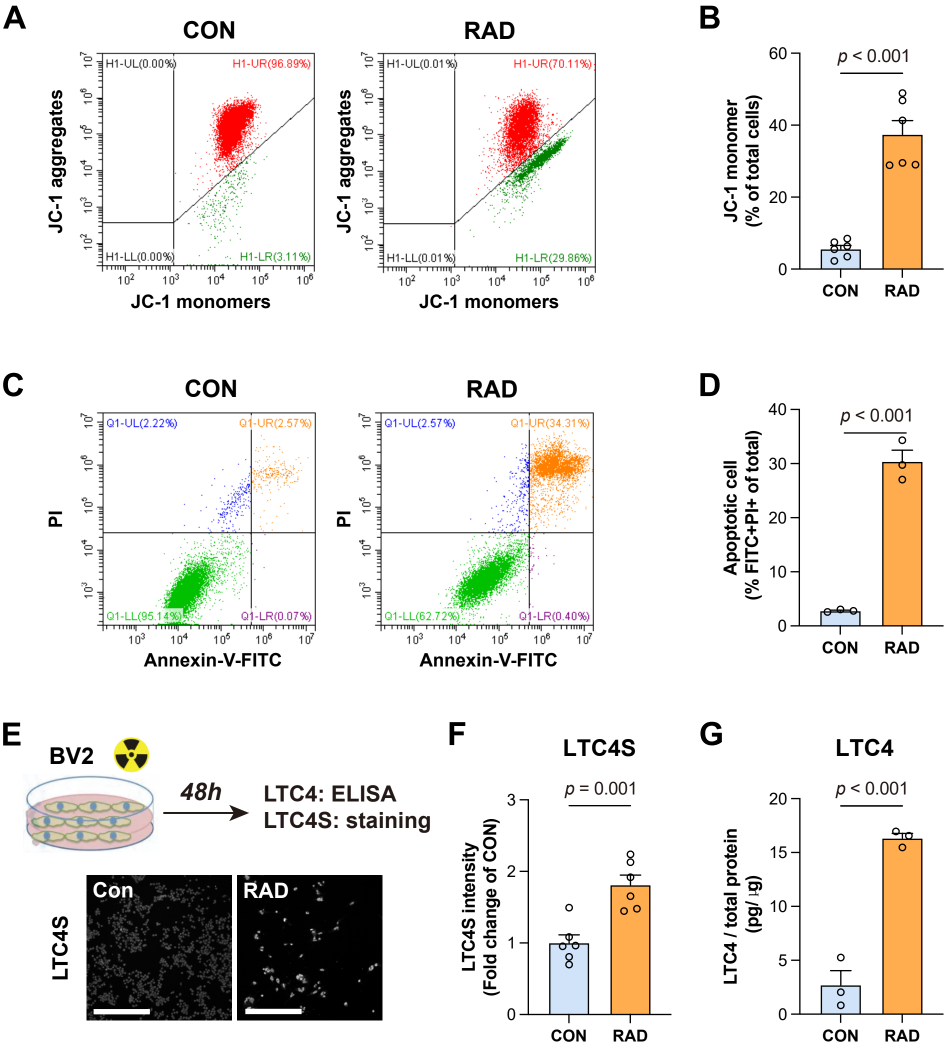
A
CON
RAD
JC-1 aggregates
JC-1 monomers
H1-UL(0.00%)
H1-UR(96.89%)
H1-LL(0.00%)
H1-LR(3.11%)
H1-UL(0.01%)
H1-UR(70.11%)
H1-LL(0.01%)
H1-LR(29.86%)
B
p < 0.001
JC-1 monomer (% of total cells)
CON
RAD
C
CON
RAD
PI
Annexin-V-FITC
Q1-UL(2.22%)
Q1-UR(2.57%)
Q1-LL(95.14%)
Q1-LR(0.07%)
Q1-UL(2.57%)
Q1-UR(34.31%)
Q1-LL(62.72%)
Q1-LR(0.40%)
D
p < 0.001
Apoptotic cell (% FITC+PI+ of total)
CON
RAD
E
BV2
48h
LTC4: ELISA
LTC4S: staining
LTC4S
Con
RAD
F
LTC4S
p = 0.001
LTC4S intensity (Fold change of CON)
CON
RAD
G
LTC4
p < 0.001
LTC4 / total protein (pg/ μg)
CON
RAD

Supplementary Figure 4. Radiation increases cell apoptosis, LTC4S expression, and LTC4 levels in the BV2 microglial cells. (**A**) Gating strategy and representative plots of JC-1 aggregates and monomers from flow cytometry analysis, measuring mitochondrial membrane potential of BV2 microglial cells from non-irradiated group (CON) and 10 Gy irradiated group (RAD). (**B**) Quantification of JC-1 monomers as the percentage of total cells (CON: n=6; RAD: n=6). (**C**) Gating strategy and representative plots of Annexin-V-FITC/PI flow cytometry analysis of BV2 microglial cells of non-irradiated group (CON) and 10 Gy irradiated group (RAD). Apoptotic cells (Annexin-V-FITC$^+$ and PI$^+$) were presented in upper right quadrant. (**D**) Quantification of apoptotic cells (Annexin-V-FITC$^+$ and PI$^+$) of total cells (CON: n=3; RAD: n=3). (E) Upper panel: schematic of the experimental designs. Lower panel: representative images of LTC4S staining of BV2 microglia cells from non-irradiated group (CON) and 10 Gy irradiated group (RAD). Scale bar: 300μm. (F) Quantification of LTC4S fluorescent intensity in irradiated BV2 microglial cells (RAD), compare to non-irradiated (CON) group (CON: n=6; RAD: n=6). (G) Quantification of the LTC4 levels in BV2 microglial cells between groups (CON: n=3; RAD: n=3). Data are represented as mean ± s.e.m. and analyzed by Student's *t* test.
